# Urbanization Reduces Transfer of Diverse Environmental Microbiota Indoors

**DOI:** 10.3389/fmicb.2018.00084

**Published:** 2018-02-05

**Authors:** Anirudra Parajuli, Mira Grönroos, Nathan Siter, Riikka Puhakka, Heli K. Vari, Marja I. Roslund, Ari Jumpponen, Noora Nurminen, Olli H. Laitinen, Heikki Hyöty, Juho Rajaniemi, Aki Sinkkonen

**Affiliations:** ^1^Ecosystems and Environment Research Programme, Faculty of Biological and Environmental Sciences, University of Helsinki, Lahti, Finland; ^2^School of Artitechture, Tampere University of Technology, Tampere, Finland; ^3^Division of Biology, Kansas State University, Manhattan, KS, United States; ^4^Department of Virology, Faculty of Medicine and Life Sciences, University of Tampere, Tampere, Finland; ^5^Fimlab Laboratories, Pirkanmaa Hospital District, Tampere, Finland

**Keywords:** urbanization, urban microbiome, indoor microbiome, built environment microbiome, environmental microbiome, soil microbiome, land-use

## Abstract

Expanding urbanization is a major factor behind rapidly declining biodiversity. It has been proposed that in urbanized societies, the rarity of contact with diverse environmental microbiota negatively impacts immune function and ultimately increases the risk for allergies and other immune-mediated disorders. Surprisingly, the basic assumption that urbanization reduces exposure to environmental microbiota and its transfer indoors has rarely been examined. We investigated if the land use type around Finnish homes affects the diversity, richness, and abundance of bacterial communities indoors. Debris deposited on standardized doormats was collected in 30 rural and 26 urban households in and near the city of Lahti, Finland, in August 2015. Debris was weighed, bacterial community composition determined by high throughput sequencing of bacterial 16S ribosomal RNA (rRNA) gene on the Illumina MiSeq platform, and the percentage of four different land use types (i.e., built area, forest, transitional, and open area) within 200 m and 2000 m radiuses from each household was characterized. The quantity of doormat debris was inversely correlated with coverage of built area. The diversity of total bacterial, Proteobacterial, Actinobacterial, Bacteroidetes, and Firmicutes communities decreased as the percentage of built area increased. Their richness followed the same pattern except for Firmicutes for which no association was observed. The relative abundance of Proteobacteria and particularly Gammaproteobacteria increased, whereas that of Actinobacteria decreased with increasing built area. Neither Phylum Firmicutes nor Bacteroidetes varied with coverage of built area. Additionally, the relative abundance of potentially pathogenic bacterial families and genera increased as the percentage of built area increased. Interestingly, having domestic animals (including pets) only altered the association between the richness of Gammaproteobacteria and diversity of Firmicutes with the built area coverage suggesting that animal ownership minimally affects transfer of environmental microbiota indoors from the living environment. These results support the hypothesis that people living in densely built areas are less exposed to diverse environmental microbiota than people living in more sparsely built areas.

## Introduction

The United Nations estimates that over half of the world's population are urban inhabitants, and by 2050, the number is expected to rise to more than two-thirds (United Nations, 2014). Forests and natural grasslands are either lacking or rare in urban areas, which limits urban dwellers' access to non-built, natural areas. This can be a consequence of major changes in land use (Butchart et al., 2010). Due to these changes and other anthropogenic disturbances, natural biodiversity in urban areas is often low (Chapin et al., 2000; Hanski et al., 2012; Thapa et al., 2017). This in turn can lead to a reduced exposure to environmental microbiota among urban inhabitants (von Hertzen and Haahtela, 2006). This effect is significant in the light of the widely recognized “hygiene hypothesis” and the “biodiversity hypothesis” which state that reduced exposure to natural microbial communities increases the risk of immune-mediated non-communicable diseases (Strachan, 1989; Haahtela et al., 2015).

The predominant routes by which humans are exposed to environmental microbes indoors include human and animal transfer of microbes from outdoors, bioaerosol transfer through open windows and doors as well as non-filtered ventilation (Hospodsky et al., 2012; Qian et al., 2012; Adams et al., 2015). Circumstantial evidence, such as changes in bacterial diversity in the near-surface atmosphere across different land use types (Shaffer and Lighthart, 1997; Burrows et al., 2009; Bowers et al., 2011) and associations between indoor bacterial communities and outdoor environmental factors (Ege et al., 2011; Barberán et al., 2015; Dannemiller et al., 2016) support the notion that exposure to environmental microbiota in urban households is limited. However, hardly any research has focused on the transfer of environmental microbiota from outdoors to indoors.

The scarcity of evidence demonstrating indoor exposure to environmental microbiota has become a fundamental issue as urban dwellers spend the majority of their time indoors (World Health Organization, 2005; Franklin, 2007). Previous studies have tried to address this issue by analyzing the household dust (or indoor bioareosols) to characterize the indoor microbial communities and their association with human health (e.g., Ross et al., 2000; Pakarinen et al., 2008; Dunn et al., 2013; Barberán et al., 2015; Dannemiller et al., 2016) with studies suggesting that the microbial community in indoor dust depends on the land use outside (Alenius et al., 2009; Ege et al., 2011). However, the use of indoor dust presumably distinguishes indoor microbial communities poorly from those transferred from the surrounding environment and the diversity of indoor bacteria is mostly influenced by human occupants (Täubel et al., 2009). The humans and pet-assisted transfer of environmental microbes indoors requires qualitative and quantitative information on the environmental litter carried inside. Since the quantity of environmental litter carried inside and its microbial composition depends mostly on the characteristics of surrounding environment, it is reasonable to assume that the indoor transfer of and exposure to environmental microbiota depends on land use type outside, which however currently remains *terra incognita*.

To fill this gap in knowledge, we investigated how different land use types (forests, open green areas, transitional areas, and built environment) affect the amount of environmental litter carried inside homes as well as the bacterial communities in the doormat debris. The study subjects were aging people that originally represented middle-aged and young pensioners in a well-being study in Päijät-Häme region, Southern Finland (Fogelholm et al., 2006). We hypothesized that the quantity of debris deposited on the doormats was inversely correlated with the coverage of built environment and that the diversity and richness of the total bacterial community in the doormat debris follow similar association. Additionally, we hypothesized that the microbial community composition in the doormat debris (including the diversity, richness, and the relative abundance of the major bacterial phyla i.e., Actinobacteria, Bacteroidetes, Firmicutes, and Proteobacteria, particularly Gammaproteobacteria) depended on the coverage of built area in the vicinity of the homes. These bacterial phyla were chosen since they are the most abundant environmental bacterial phyla and they have been observed to be associated with immune system modulation as demonstrated by previous studies (Pakarinen et al., 2008; Round and Mazmanian, 2010; Fahlén et al., 2012; Hanski et al., 2012). As natural environments are likely to host more diverse microbiota than built environment, we assumed that some operational taxonomic units (OTUs) at genus level were indicators of non-built environment, while no or only few OTUs were indicators of built environment. Finally, we assumed that confounding factors such as owning domestic animals hardly affected the observed associations.

## Materials and methods

### Study sites

**Figure 1:**
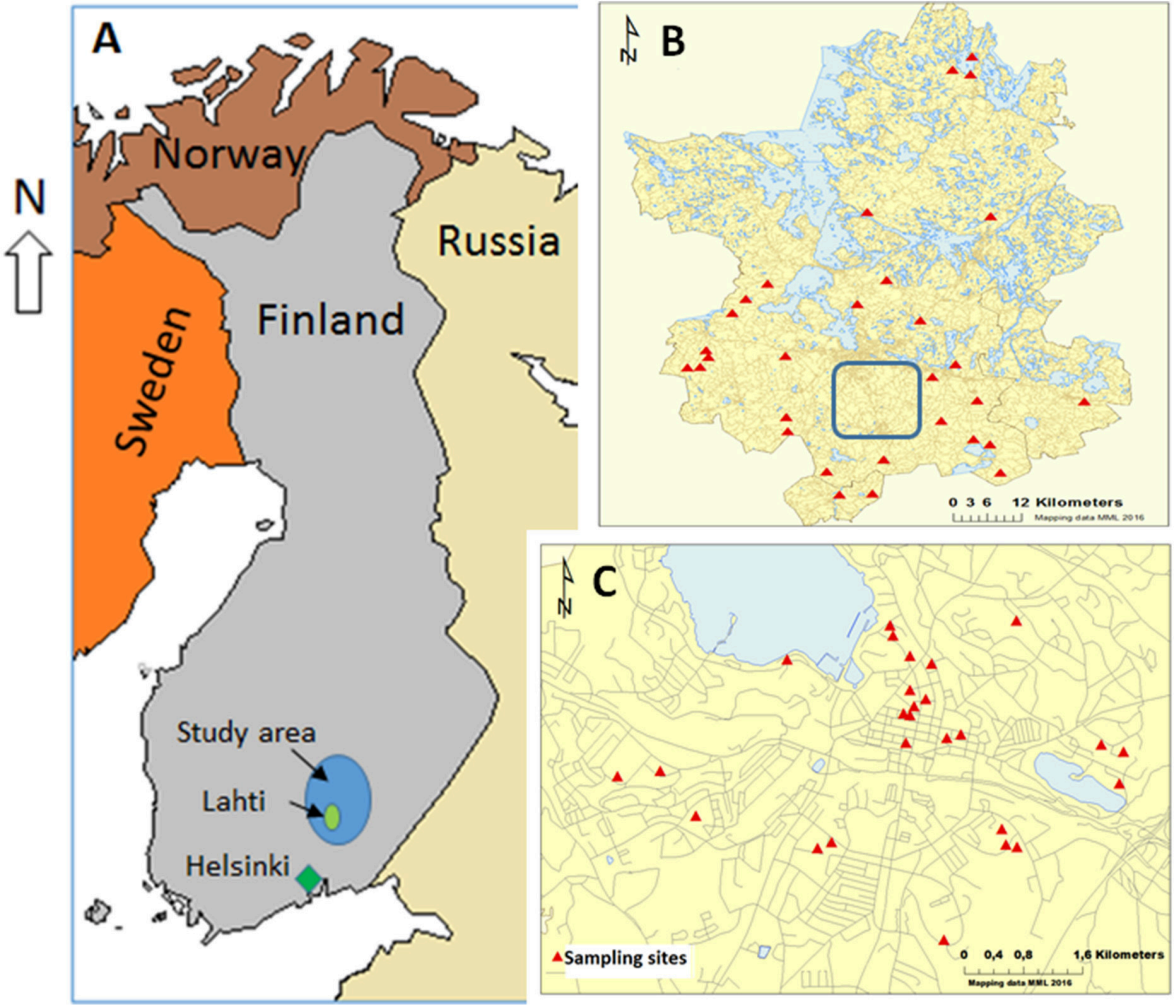
The study sites included in the study. **(A)** The study area located in Finland. **(B)** The rural sampling sites (indicated by red triangles). **(C)** The urban sampling sites in the city of Lahti (enclosed with the blue inset box in **B**). A total of 26 sites from the urban area and 30 sites from the rural area were included in the study.

The study subjects were selected among the participants of a large 10-year (2002-2012) prospective study *GOAL—Good Aging in Lahti region* that aimed to find associations between the living environment (rural, urban, and semi-urban) and any chronic diseases and functional disabilities in elderly and retired population (Fogelholm et al., 2006). The study subjects' homes were chosen as research sites in the current study, and they were located within the city of Lahti and in the surrounding countryside that included the region of Päijät-Häme and two other municipalities (Iitti and Pukkila) in Southern Finland (Figure 1). A total of 56 sites were included in the study. Twenty six of those were located within the city limits of Lahti that has 100,000 inhabitants and a population density of over 700 inhabitants per sq. km (referred to as the urban sites afterwards). All urban sites comprised study subjects' homes in apartment buildings in densely populated areas in Lahti. The remaining 30 sites were located in sparsely populated rural areas around Päijät-Häme and its surroundings (referred as the rural sites from now on) comprising participants' homes in farmhouses (active or non-active) or other detached houses. According to the Nordic standard, sparsely populated areas are located outside “densely populated areas” (“taajama” in Finnish) in which buildings are maximum of 200 m apart and include at least 200 inhabitants. In municipalities where the rural sites were located, population density was less than 50 inhabitants per sq. km (Päijät-Hämeen verkkotietokeskus, 2011). The characteristics of urban and rural participants of the study are summarized in Table 1.

**Table 1:** Characteristics of urban and rural participants of the study.

	**Urban participants**	**Rural participants**
**SEX**		
Female	11	15
Male	15	15
**AGE COHORT**		
65–69 years	12	17
75–79 years	14	13
**FAMILY**		
Living alone	10	8
Living with family member(s)	16	22
**DOMESTIC ANIMALS (INCL. PETS)**		
Indoors	3	7
Only outdoors	0	7

This study was carried out in accordance with the recommendations of the “*Finnish Advisory Board on Research Integrity*” with an approval from the ethical committee (*Tampereen yliopistollisen sairaalan erityisvastuualueen alueellinen eettinen toimikunta*). Written informed consents were obtained from all subjects that were in accordance with the *Declaration of Helsinki*.

### Doormat installation, collection, and recovery of materials

The polyethylene doormats used in the study were scraper plastic mats (dimensions of 45 × 57 cm) that effectively collect dirt and matter. All participants were delivered a new, unused doormat which was placed indoors immediately at the main entrance door of the study participant's home for an average of 18 (± 2) days (August 2015) to gather material carried inside their home. The participants were instructed to wipe their shoes after stepping on the mat when they entered their home and not to clean the mat during the usage period. The material deposited in the mat was collected after the study period on site. First, any loose organic matter such as leaves, twigs, and other large plant parts were transferred into a sealable freezer bag by hand. The mat was then turned upside down on a clean aluminum foil and tapped all over for about 10 s to collect all residual material. The material on the foil was collected into a plastic bag. Clean, disposable laboratory gloves were used when collecting the samples. The bag was sealed airtight and frozen immediately on dry ice. The sample weights were calculated by subtracting the weight of the empty plastic bag from the total weight that was weighed on a standard laboratory balance.

### DNA extraction, amplification, and sequencing

#### Sample preparation for MiSeq sequencing

Samples for MiSeq sequencing were prepared according to Veach et al. (2015). Total DNA was extracted using PowerSoil® DNA Isolation Kit (MoBio Laboratories, Inc., Carlsbad, CA, USA) according to the manufacturer's standard protocol from 0.25 ± 0.07 g (target amount ± *SD*) of doormat debris in three separate replicates. Sterile water (250 μl) was used as a negative control. The quality of the extracted DNA was checked using agarose gel (1.5%) electrophoresis and quantified with Quant-iT™ PicoGreen® dsDNA reagent kit (Thermo scientific, MA, USA). The DNA concentration was adjusted to 0.35–0.4 ng/μl for each sample.

DNA was analyzed for bacterial (16S rRNA gene) communities using a two-step PCR approach to avoid 3′-end amplification bias resulting from the sample-specific DNA tags (Berry et al., 2011). The V4 region within the 16S ribosomal RNA (rRNA) gene was amplified in triplicates during the primary PCRs using 515F and 806R primers (Caporaso et al., 2011, 2012). The primary PCRs were carried out in 50 μl reaction volumes consisting of 1 μl each of 10 mM deoxynucleotide triphosphates (dNTPs; Thermo scientific, MA, USA), 5 μl forward primer 505F (10 μM; 5′ –GTGCCAGCMGCCGCGGTAA-3′) and 5 μl reverse primer 806R (10 μM; 5′-GGACTACHVGGGTWTCTAAT-3′), 0.5 μl 2 U/μl Phusion Green Hot Start II High-Fidelity DNA polymerase (Thermo scientific, MA, USA), 10 μl 5x Green HF PCR buffer (F-537), 5 μl of template DNA, and 23.5 μl sterile water. The PCR reactions were run in thermocycler (MJ Research, MA, USA) as follows: initial denaturation at 98°C for 5 min followed by 25 cycles of denaturation at 94°C for 1 min, annealing for 10 s at 50°C, extension for 1 min at 72°C, and then a final extension at 72°C for 10 min. A positive control (*Cupriavidus necator* JMP134, DSM 4058) was included in all PCRs, and sterile water was again used as a negative control to detect possible contaminations. The PCR products were visualized using agarose gel (1.5%) electrophoresis. The PCR products were purified using Agencourt AMPure XP solution (Beckman Coulter Inc., 1:1 ratio of bead solution to PCR volume) and eluted with sterile water to minimize the carryover of primary PCR primers. Triplicates of the cleaned amplicons were pooled and diluted with sterile water in 1:5 ratio.

Cleaned and diluted primary PCR products were targeted in the secondary PCR (TagPCR). The reaction mixture of the TagPCR was the same as above with the exception of the reverse primer which included a 12 bp unique Multiplexing Identifier tag (MID-806R). Amplification reactions were identical to those for primary PCRs except that they included only 10 cycles of denaturation. TagPCR products were visualized on agarose gel (1.5%) electrophoresis, purified with Agencourt AMPure after which the triplicate amplicons were pooled. DNA concentration of each sample was measured using Quant-iT™ PicoGreen®, and pooled samples had equal amounts of DNA (150 ng). The GeneRead DNA Library I Core Kit (Qiagen, catalog # 180432) was used to ligate Illumina's TruSeq adapters to amplicons. The sequencing was performed in the Integrated Genomics Facility (http://www.k-state.edu/igenomics/) at Kansas State University using Illumina MiSeq platform with a 2 × 300 bp version 3 sequencing kit according to manufacturer's protocol.

#### Sequence processing

Raw sequence data were processed using Mothur (version 1.39.5, Schloss et al., 2009). The sequence processing protocol largely followed the pipeline suggested by Schloss et al. (2011) and Kozich et al. (2013). The paired sequences contained in reverse and forward fastq files were aligned into contigs. Contiged sequences were trimmed and screened to remove any mismatches to primers or DNA-tag sequences, or with ambiguous bases and homopolymers longer than 8 bp. Sequences were aligned using Mothur version of SILVA bacterial reference (version 123, Pruesse et al., 2007), and those that remained unaligned against the reference were removed. Near identical (>99% similar) unique sequences were preclustered to minimize sequencing errors (Huse et al., 2010). The data were screened for chimeras with UCHIME (Edgar et al., 2011) which uses the most abundant sequences as a reference. The chimeric sequences were removed. To reduce computing time, samples were subsampled to 20,000 sequences. Pairwise distance matrix for unique sequences was calculated and OTUs clustered at 97% sequence similarity using OptiClust (Westcott and Schloss, 2017). Sequences were classified using the Mothur version of Naive Bayesian classifier (Wang et al., 2007) with the RDP training set version 14 (Cole et al., 2009). Sequences classified as Chloroplast, Mitochondria, unknown, Archaea, or Eukaryota were removed. OTUs represented by 10 or fewer sequences in the dataset or those detected in negative controls were removed. Finally, all samples were subsampled to 2,000 sequences to normalize sequencing depth. Six out of 56 doormat samples yielded few sequences and were therefore omitted from further analyses resulting in 30 samples from rural and 20 from urban area remaining.

#### Land cover class determination

The percentages of land cover types up to 2000 m radius of the study sites were estimated using the CORINE Land Cover 2012 database. The percentages of four different land cover categories. i.e., built area (including hardscapes), open area (spaces with voluminous open nature), forest, and transitional area in the radius of 200 m and 2000 m were characterized and included in regression analyses.

### Statistical analyses

The quantity of debris in the doormat samples was not normally distributed. Accordingly, differences in the debris weight between the rural and urban doormat samples and between the two age-cohorts were inferred using the non-parametric Wilcoxon-test. OTU richness and Shannon diversity index were calculated using functions specnumber and diversity in R package *vegan* respectively. For the major bacterial phyla (and the classes under Proteobacteria), Shannon diversity index and richness were also calculated by rarefying the samples to even sampling depth. Principal Coordinate Analysis (Gower, 1966) using Bray-Curtis distance was performed using cmdscale function in R package *stats* using the function vegdist in package *vegan*. Characteristic or indicator species were identified by the indicator species analyses that combined the relative abundance with the relative frequency of occurrence of a species in the urban and rural doormat debris samples using the indval function in the R-package *labdsv* (Dufrene and Legendre, 1997). The associations between the land cover and bacterial diversity and richness of overall bacterial community as well as the diversity and richness of the major bacterial phyla together with their relative abundances were determined using multiple linear regressions in the linear model function using the package MASS (Venables and Ripley, 2002) in R (R v3.3.2, R Core Team, 2015). Regression models were based on the stepwise method that employs a combination of forward and backward elimination of explanatory i.e., land use variables. Regression models were compared using Akaike Information Criterion (AIC) and those sets of land use variables that minimized AIC were selected. Because of a high correlation (>70%) with other explanatory variables, the open area was excluded in the analyses.

### Data availability

Raw sequence data are available in Sequence Read Archive with accession number SRP115276.

## Results

### Doormat debris quantity and its relation with land use

The amount of doormat debris correlated inversely with the coverage of built area within 200 m (*R*^2^ = 0.103, *p* = 0.0096, Figure S1A) and correlated positively with the percentage of forest within the 2000 m radius of the study sites (*R*^2^ = 0.13, *p* = 0.006, Figure S1B). The weights of debris differed between the rural and urban dwellings (W = 828, *p* < 0.001), and the mean weight in the countryside (13.78 ± 17.44 g) was an order of magnitude greater than within the city (1.37 ± 2.99 g). In addition to these quantitative properties, visual inspection revealed a trend; urban sandy debris included non-organic materials such as plastics, whereas rural samples were characterized by abundant organic soil particles. The amount of litter carried inside the house did not differ between the two age cohorts i.e., between 65–69 and 75–79 years old people (W = 281, *p* = 0.55).

### Bacterial community characterization

**Figure 2:**
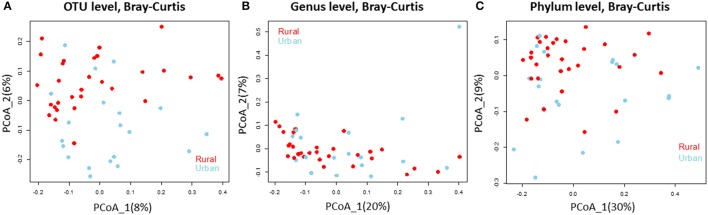
Bacterial community composition does not differ in the rural and urban doormat samples. Principal Coordinate Analysis (based on Bray-Curtis dissimilarity metric) of the bacterial samples taken from rural (red) and urban (sky blue) doormat samples at the **(A)** OTU level, **(B)** Genus level, and **(C)** Phylum level.

We analyzed the 16S rRNA amplicon dataset from the 50 samples collected from rural and urban doormat debris and obtained a total of 4797 OTUs that represented 25 known bacterial phyla. These bacterial communities represented 50 identified classes and 362 known genera. Bacterial OTUs within Bacteroidetes were the most abundant in the rural samples, accounting for 23.79% of the total sequences followed by Proteobacteria (21.30%), Actinobacteria (19.36%), and Firmicutes (8.09%). Proteobacterial OTUs were the most abundant in urban doormat samples with 23.95% of the total sequences, followed by Bacteroidetes (23.93%), Actinobacteria (15.09%), and Firmicutes (10.79%) (Table S1). A similar distribution of the major bacterial phyla was observed in the individual samples taken from both sites (Figure S2). The principal coordinate analysis revealed that the bacterial community composition did not differ between the rural and urban samples at the OTU level, genus level, or the phylum level (Figures 2A–C).

### Diversity and richness of the bacterial communities and their relation with land use

**Figure 3:**
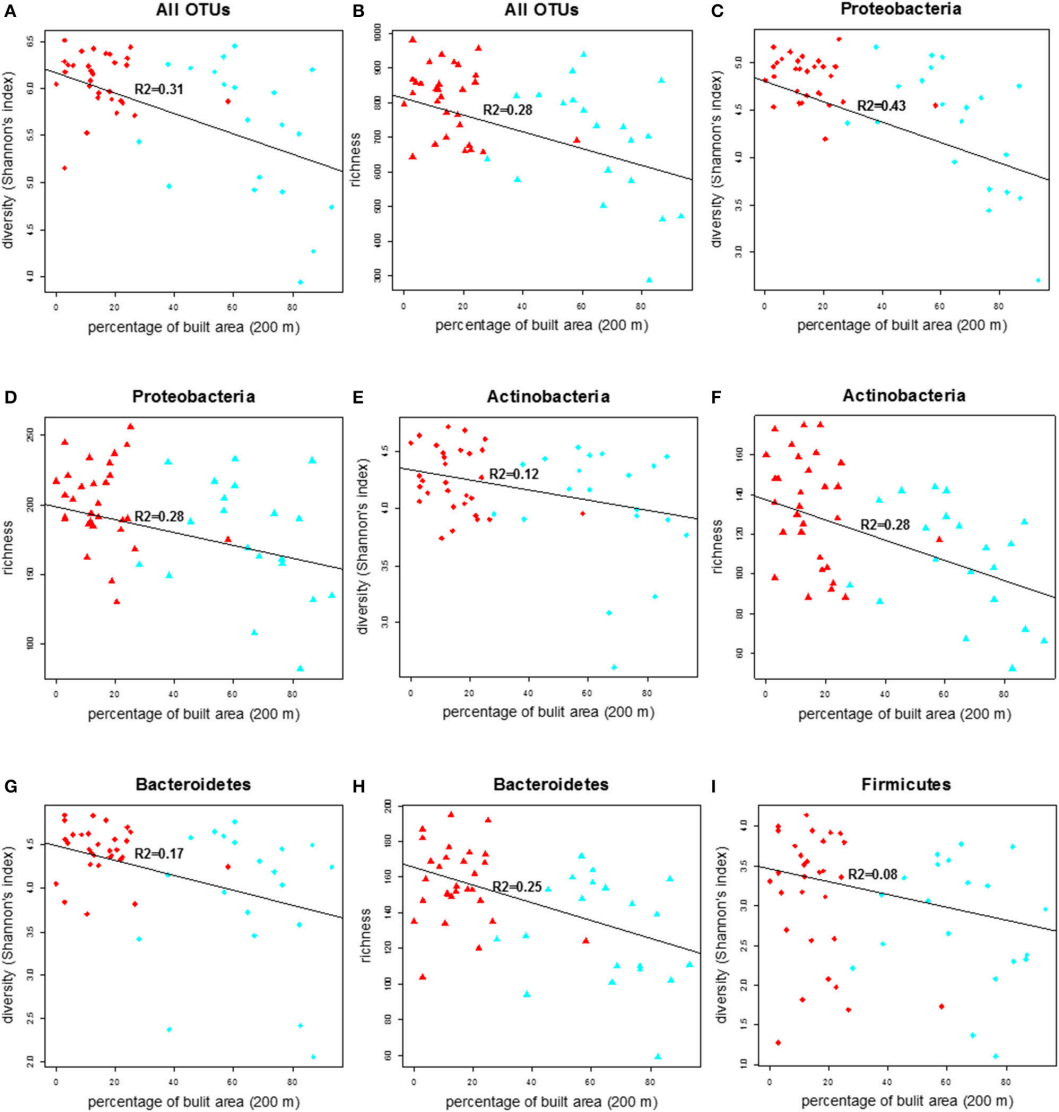
The correlation plot of diversity (Shannon index) and richness of total bacterial community and other major phyla in the doormat debris in relation to the percentage of built area within the 200 m radius of the study sites. The red diamonds and triangles in figures **(A–F)** and **(G–I)** represent rural sites and the corresponding shapes in cyan denote urban sites.

We compared the diversity of the doormat bacterial communities, measured as the Shannon's index, and the richness across the land use variables and found that the diversity and richness of the overall bacterial community declined with increasing percentage of built area within 200 m (Figures 3A,B; Table 2). No such associations were observed between the bacterial diversity and richness and other land use types, i.e., percentage of forest and transitional areas (Table S2) when the 200 m radius was used. Curiously, however, the diversity and richness of the total bacterial community increased as forest cover increased within the larger, 2000 m radius (Figures S3A–D), even though the negative associations between the coverage of built area and the diversity (*R*^2^ = 0.29, *p* < 0.001) and richness (*R*^2^ = 0.27, *p* < 0.001) of the total bacterial community were still evident (2000 m radius; Figures S3A–D).

**Table 2:** Regression analysis summary of bacterial diversity (Shannon index) and richness for the whole community and for the major bacterial phyla vs. the percentage of built area within 200 m radius of the study sites.

**Diversity**	***R*^2^**	**DF**	***t*-value**	***p*-value**
All OTUs	0.31	48	−4.64	< 0.001
Proteobacteria	0.43	48	−5.99	< 0.001
Actinobacteria	0.12	48	−2.62	0.011
Bacteroidetes	0.17	48	−3.12	0.003
Firmicutes	0.08	48	−2.05	0.045
Richness				
All OTUs	0.28	48	−4.30	< 0.001
Proteobacteria	0.17	48	−3.17	0.002
Actinobacteria	0.28	48	−4.34	< 0.001
Bacteroidetes	0.25	48	−4.05	< 0.001

The diversity and richness within the major bacterial taxa in the doormat debris generally depended on the percentage of built area within the 200 m radius. The diversity of Proteobacteria was depended strongly and inversely on the coverage of built area within the 200 m radius, while its richness had similar but weaker association (Figures 3C,D; Table 2). Similar association between the coverage of built area and diversity (*R*^2^ = 0.39, *p* < 0.001) and richness (*R*^2^ = 0.09, *p* = 0.036) was also observed for Gammaproteobacteria (Figures S4A,B; Table S2). The diversity of Alphaproteobacteria was inversely associated with the coverage of built area and directly associated with the percentage of forest (*R*^2^ = 0.21, *p* = 0.003, Figures S4C,D; Table S2), while its richness was associated negatively with the percentage of built area within the 200 m radius (*R*^2^ = 0.11, *p* = 0.016, Figure S4E; Table S2). The diversity of Betaproteobacteria showed negative association with the percentage of built area and the percentage of transitional area (*R*^2^ = 0.23, *p* = 0.001, Figures S4F,G; Table S2), and its richness had negative association with the percentage of built area within the 200 m radius (*R*^2^ = 0.12, *p* = 0.011, Figure S4H; Table S2). The diversity and richness of Actinobacteria and Bacteroidetes had strong and negative associations with the coverage of built area within 200 m (Figures 3E–H; Table 2). The association between the diversity of Firmicutes and the built area coverage was significant but weak (Figure 3I; Table 2), and no association was observed for its richness.

Removal of households having domestic animals (including pets) from the data did not alter the findings with the exception of Firmicutes and Gammaproteobacteria. In the absence of animals, neither diversity nor richness of Firmicutes was associated with the coverage of built area within 200 m. In case of Gammaproteobacteria, the diversity was still strongly and directly dependent on the built area coverage, while the richness became independent (Table S3).

We also investigated the association between the diversity and richness of the individual bacterial taxa and land coverage by rarefying the sequence depth (Table S4). We observed that the diversity (*R*^2^ = 0.41, *p* < 0.001) and richness (*R*^2^ = 0.38, *p* < 0.001) of Proteobacteria had strong and inverse relationship with the percentage of built area. Inverse but weaker associations were observed between the percentage of built area and the diversity and richness of Alphaproteobacteria [*R*^2^ = 0.16, *p* = 0.003, (diversity), *R*^2^ = 0.19, *p* = 0.001 (richness)], Betaproteobacteria (*R*^2^ = 0.23, *p* = 0.012 (diversity), R^2^ = 0.25, *p* = 0.005 (richness)], and Gammaproteobacteria [*R*^2^ = 0.32, *p* < 0.001 (diversity), *R*^2^ = 0.28, *p* < 0.001 (richness)]. The inverse association between the percentage of built area and diversity (*R*^2^ = 0.26, *p* < 0.001) and richness (*R*^2^ = 0.26, *p* < 0.001) of Bacteroidetes was relatively weak but highly significant. The richness of Firmicutes exhibited weak and inverse asscociation with the percentage of built area (*R*^2^ = 0.08, *p* = 0.04), while no association was observed for its diversity. Likewise, there was no association between land coverage and diversity and richness of Actinobacteria (Table S4).

### Relative abundances of bacterial taxa and their associations with land use

Most of the major phyla (e.g., Bacteroidetes and Firmicutes) did not have any association with any of the land cover types (Table S5), but the relative abundance of Proteobacteria increased with increasing percentage of built area (*R*^2^ = 0.11, *p* = 0.017, Figure S5A, Table S5). Gammaproteobacteria had a similar but stronger association (*R*^2^ = 0.23, *p* < 0.001, Figure S5B, Table S5). The relative abundance of Betaproteobacteria had a weak positive association with the percentage of transitional area (*R*^2^ = 0.09, *p* = 0.027, Table S5) and that of Alphaproteobacteria did not respond to any of the land use variables (Table S5). In contrast to Proteobacteria, the relative abundance of Actinobacteria decreased as the built area increased (*R*^2^ = 0.24, *p* < 0.001, Figure S5C, Table S5). At the class level, the relative abundance of Actinobacteria (*R*^2^ = 0.23, *p* < 0.001) and Acidobacteria Group 16 (*R*^2^ = 0.08, *p* = 0.04) declined with the increase in coverage of built area. Likewise, the relative abundance of order Actinomycetales (*R*^2^ = 0.21, *p* < 0.001), Bacillales (*R*^2^ = 0.22, *p* < 0.001), Solirubrobacterales (*R*^2^ = 0.10, p = 0.02), Rhodobacterales (*R*^2^ = 0.22, *p* < 0.001), Rubrobacterales (*R*^2^ = 0.12, *p* = 0.01), and Acidobacterales Group 16 (*R*^2^ = 0.07, *p* = 0.04), decreased while that of Enterobacterales (*R*^2^ = 0.12, *p* = 0.01) increased with the increase in built area. At the family level, the relative abundance of Streptococcaceae (*R*^2^ = 0.10, *p* = 0.02), Enterobacteriaceae (*R*^2^ = 0.13, *p* = 0.01), and Mycobacteriaceae (*R*^2^ = 0.10, *p* = 0.04) exhibited positive association and that of the families Pseudomonadceae (*R*^2^ = 0.22, *p* = 0.001), Solirubrobacteraceae (*R*^2^ = 0.12, *p* = 0.01), Rhodobacteraceae (*R*^2^ = 0.22, *p* < 0.001), Planococcaceae (*R*^2^ = 0.17, *p* = 0.005), and Intrasporangiaceae (*R*^2^ = 0.31, *p* < 0.001) revealed negative association with the coverage of built area within 200 m radius. At the genus level, the relative abundance of Streptococcus (*R*^2^ = 0.11, *p* = 0.01) and Mycobacterium (*R*^2^ = 0.10, *p* = 0.049) was directly associated and that of Pseudomonas (*R*^2^ = 0.11, *p* = 0.017), Solirubrobacter (*R*^2^ = 0.12, *p* = 0.01), and Arsenicoccus (*R*^2^ = 0.16, *p* = 0.03) was inversely associated with the coverage of built area (Table S6). Other genera had no association or had too low total abundance with several zero abundances across samples compromising reliable statistical analyses. The relative abundance of Actinobacteria and Gammaproteobacteria followed the same pattern while that of Proteobacteria was independent of the coverage of built area within a 200 m radius around the participants' homes when pet owners were removed from the data (Table S7).

### Indicator species analysis

Indicator species analysis revealed 246 OTUs associated with either rural or urban doormat samples (Table S8). Most of these OTUs (151 in total) indicated rural environment with the OTUs representing Bacteroidetes and Proteobacteria being the most frequent (33 each), followed by Actinobacteria that had 31 representative OTUs and 9 OTUs belonged to Firmicutes. 22 of those indicator OTUs were unclassified. Out of the 151 OTUs that were indicators of rural environment, 32 of them were detected exclusively from rural doormat samples. Eight of those 32 indicator OTUs belonged to the phylum Bacteroidetes, 7 OTUs represented Proteobacteria, 5 were classified as Actinobacteria and 6 of them were unclassified bacterial phyla and only one OTU within Firmicutes exclusively represented rural sites. Out of the 95 OTUs representing urban environment, Proteobacteria was the most abundant phyla with 25 representative OTUs, followed by Bacteroidetes (23), Actinobacteria (11) and Firmicutes (9), while 15 OTUs were unclassified. 13 OTUs represented solely urban doormat samples with 5 of them belonging to Bacteroidetes, 2 representing Firmicutes and Actinobacteria and a single Proteobacterial OTU. Two of the OTUs were unclassified (Table S8). However, most of these indicator OTUs had a relatively low frequency and low abundance. Therefore an arbitrary mean value of at least three OTUs per sample was defined to identify strong indicators of either rural or urban environment. From the 246 indicator OTUs, only 29 OTUs that indicated rural doormats and five OTUs that indicated urban doormats reached the criterion. Four of the urban OTUs belonged to Gammaproteobacteria and one was a species of Streptococcus. Strong indicators of rural doormat bacterial communities consisted of 8 Actinobacterial, 7 Bacteroidetes, 2 Firmicutes OTUs and a single Acidobacterial, Alphaproteobacterial, and Gammaproteobacterial OTU as well as an unclassified OTU. Within the 8 Bacteroidetes OTUs, 3 OTUs belonged to *Hymenobacter* ssp. and within Actinobacteria two OTUs belonged to *Solirubrobacter* ssp.

## Discussion

In this study, we investigated how land cover classes are associated with the quantity as well as the qualitative (diversity, richness, and relative abundance of the overall bacterial community and major bacterial taxa) variables of the debris deposited on the experimental doormats. As an approval to our first hypothesis, we observed that people living in urban, densely built environment carry remarkably less litter home than do people who live in countryside or areas characterized by high percentage of green areas, such as forests and agricultural fields. This is an important finding as soil is a major reservoir of environmental microbes and as the amount of doormat debris in our opinion is the best estimate of soil carried inside. Further, doormat debris in sparsely built environment typically included fine organic particles, e.g., garden soil, whereas that in densely built environment contained large more inorganic particles such as gravel and pieces of plastic (see Figure S6). The human immune system is naturally modulated to distinguish pathogens and other microbes from harmless environmental particles and human tissues. Therefore, our finding suggests that people in sparsely built, mostly rural areas possibly carry more immunomodulatory organic particles, such as bacteria, indoors than people living in densely built, i.e., urban areas. An increased amount of soil and plant material carried inside, therefore can be expected to cause an enhanced exposure to environmental microbes, which might have a positive effect on human immune system and health.

These data strongly support our second main hypothesis that the microbial community composition in the doormat debris depends on the land use in the immediate vicinity of resident's home. Our study demonstrated that reduced direct contact with non-built green environment caused by expanding urbanization leads to reduced exposure to diverse environmental microbiota indoors. These findings fill an important gap identified in the review by Wills-Karp et al. (2001) who concluded that urbanization limits the exposure to diverse environmental microbial communities that are beneficial to human health. We observed that the diversity and richness of total bacterial, Proteobacterial, Actinobacterial, and Bacteroidetes communities in the doormat debris declined with the increase in built area coverage within 200 m of the participants' houses. The associations did not change substantially even after removing the animal owners from the data. In addition, *Hymenobacter* sp. and *Solirubrobacter* sp. that have been previously isolated from grass and forest soils, e.g., from burrows of earthworms *Lumbricus rubellus* (Singleton et al., 2003; Kim et al., 2008; Jin et al., 2014), were identified as indicators of rural environment.

Interestingly, the strong and significant effect of built environment on doormat Gammaproteobacterial community and the slight but significant effect on doormat Firmicutes community disappeared after removing the animal owners from the data. Firmicutes are one of the two dominating phyla in the gut (Ley et al., 2006; Zhu et al., 2010) and rare or even absent in surface soils, except in composts containing animal dung (Yu et al., 2015; Mehta et al., 2016), possibly explaining this outcome. Notably, we did extensive literature review to search for the origin of most common 30 Firmicutes OTUs that represented 50% of total Firmicutes OTUs (data not shown). In addition to eight unidentified OTUs, these OTUs belonged to genera Lactobacillus (5 OTUs), Streptococcus (2 OTUs), Lactococcus (2 OTUs), Enterococcus (2 OTUs), Blautia (2 OTUs), Roseburia (2 OTUs), Faecalibacterium (1 OTU), Staphylococcus (1 OTU), Megamonas (1 OTU), Subdoligranulum (1 OTU), Finegoldia (1 OTU), and Leuconostoc (1 OTU). These bacterial genera are commensal flora of skin, gut, upper respiratory tract and salivary and mucosal membrane in humans and animals (Kloos, 1980; Hammes and Vogel, 1995; Patterson, 1996; Devriese et al., 2006; Casalta and Montel, 2008; Fournier et al., 2008; Zheng et al., 2009; Rajilić-Stojanović and de Vos, 2014; de Steenhuijsen Piters et al., 2015). Thus, the probable reason for the lacking effect of built environment on Firmicutes doormat community *per se* is direct and human assisted transfer of gut microbiota from animals onto the doormats mainly through foot wears.

In contrast, the associations between the Gammaproteobacterial doormat community and the pets seem to be rather complicated. The diversity of the Gammaproteobacterial community decreased as the percentage of built environment increased. However, when animal owners were removed from the data, the relationship between the percentage of built environment and richness of Gammaproteobacterial community disappeared whereas the diversity still had an inverse association with the percentage of built area. The most plausible explanation for this is that the pet owners accessed various sources of Gammaproteobacteria more easily than other study participants. The pet owners and their doormats may have obtained the diverse indigenous Gammaproteobacterial communities originating from their animals. The owners or their pets may have visited habitats commonly occupied by Gammaproteobacteria, such as puddles and wetlands that are intentionally or subconsciously avoided by aging people without pets. This is an interesting possibility as members of Gammaproteobacteria can have a role in prevention of atopy and allergic sensitization (Hanski et al., 2012; Haahtela et al., 2015). Our findings highlight the importance of easy access to natural microbiota because limited exposure to microbes can contribute to the risk of immune-mediated and other non-communicable diseases at the population level (Bach, 2002; Viinanen et al., 2005; von Mutius and Vercelli, 2010; Ege et al., 2011; Graham-Rowe, 2011; Rook, 2013).

In parallel to analyzing the association between urbanization and diversity discussed above, we investigated the differences in the relative abundance of bacterial taxa detected in doormat samples in response to increasing urbanization. At the phylum level, relative abundance of Actinobacteria had a strong inverse and Proteobacteria had a relatively weak but direct association with the coverage of built area. Interestingly, the relative abundance of Bacteroidetes and Firmicutes, that are the two major phyla in the human and animal guts, were not dependent on the percentage of built environment, even though the diversity and richness of particularly Bacteroidetes decreased as the level of urbanization increased. This may be due to more simpler communities in urban settings as a result of strains originating from humans and human activities replacing those originating in the green environment. As the relative abundance of Actinobacteria had a strong negative association with urbanization, regardless of whether or not the animal owners were included, we propose that aging people living in densely built urban environments are infrequently exposed to natural and diverse Actinobacterial communities.

We also observed that the relative abundances of Proteobacteria and its class Gammaproteobacteria increased as the percentage of built area increased. Interestingly, the exclusion of animal owners did not alter the Gammaproteobacterial association while the association at the level of the phylum Proteobacteria disappeared. As most of the animal owners in our study lived in sparsely built areas, it seems that in countryside daily contacts between humans and animals, or between animals and the doormats, increase the relative abundance of non-Gammaproteobacterial taxa on doormats, i.e., the transfer of non-Gammaproteobacterial microbiota indoors. Other alternatives include the possibility of competition between the microbes on the doormats, and the role of pets in enriching those that persist. As doormat debris typically has a large surface area, it likely looses moisture quickly, ceasing active microbial growth. In addition, as five Gammaproteobacterial OTUs indicated urban environment, it seems likely that certain members of Gammaproteobacteria are more abundant in urban than in rural doormats. The explanation for this Gammaproteobacterial characteristics remains a mystery as our study was not intended to reveal doormat community dynamics but to provide information on how green cover and the percentage of built environment is associated with doormat bacterial communities.

Our findings on the increased (Gamma)Proteobacterial and decreased Actinobacterial presence on urban doormats are surprisingly similar to earlier observations by Pakarinen et al. (2008) who reported that the relative abundance of Proteobacteria is higher and Actinobacteria is lower in the household dust of atopic individuals from the Karelia region of Finland and Russia (Haahtela et al., 2015). In addition, there is evidence that urbanization is associated with an increased risk for immune-mediated diseases including psoriasis (Yemaneberhan et al., 1997; Viinanen et al., 2005; von Hertzen and Haahtela, 2006; Weinmayr et al., 2007; Gao et al., 2008; Schröder et al., 2015) and that Actinobacteria are underrepresented in psoriatic patients (Gao et al., 2008; Fahlén et al., 2012). These observations are important in the context of our study for three reasons. First, Actinobacteria are dominant organisms in surface soils (Jiang et al., 2006). Secondly, in our analysis, nine OTUs in Actinomycetales were strong indicators (*p* ≤ 0.01) for sparsely built environments. Seven out of these nine OTUs were found in at least two thirds of rural doormats, whereas only one OTU in Actinomycetales, a Mycobacterium, was an urban environment indicator as it was found in low numbers in 16 urban and 12 rural doormats. Thirdly, Gao et al. (2008) reported that *Pseudomonas* sp. was one of the key indicators of healthy skin and in our study the relative abundance of the genus Pseudomonas declined with an increase in the coverage of built area and 2 *Pseudomonas* OTUs indicated rural environment (Table S6), both of which were present in circa one third of the rural households. Obviously, genus-level associations are arguably anecdotal, but the similarities between our analysis and the study by Gao et al. (2008) are intriguing. These results highlight the need for further research on whether and how environmental Gammaproteobacteria and Actinomycetales are associated with urbanization and human health.

A detailed analysis at the family and genus level revealed that the relative abundance of three potentially pathogenic bacterial families Enterobacteriaceae, Streptococcaceae (including the genus Streptococcus), and Mycobacteriaceae (and the genus Mycobacterium) had weak but significant direct association with increasing urbanization. The first 2 families include the most common pathogenic genera and species (Schwaber and Carmeli, 2008; Lory, 2014) and the genus Mycobacterium comprise of well-known and highly pathogenic species (Bottai et al., 2014). Our results thus indicated that urban inhabitants were exposed to less diverse environmental microbiota and to potentially pathogenic bacteria at the same time. Moreover, as reported in our previous study (Parajuli et al., 2017), environmental pollutants such as polyaromatic hydrocarbons (PAH) can change bacterial communities in an urban context. Therefore, our findings suggest that anthropogenic disturbances are responsible for the difference in microbial community composition between rural and urban settings.

We also searched for common taxa between our indicator species analysis and earlier findings by Barberán et al. (2015) who surveyed home dusts in the USA and Mhuireach et al. (2016) who explored airborne bacterial communities and urban greenness. We found only a single overlapping genus: *Porphyromonas* spp. were more common in household dust found in homes of dog owners in the study of Barberán et al. (2015), and also slightly indicated urban environment in our study. As none of our urban dwellers with *Porphyromonas* sp. had a dog, differences between the two studies may explain the opposite results. For instance, we studied indoor doormats of aging people in a region in Southern Finland, while Barberán et al. (2015) conducted a continent wide exploration targeting indoor dust in a broad range of home designs. The lack of similarities between the earlier studies and ours underline the need to fill the gaps in current knowledge on the transfer of environmental bacteria indoors. However, the differences could also be explained by the differences in the confounding factors such as the extent of outdoor activities. In our study, we did not ask the participants to keep track of the number of times they entered the house, but we are confident that most of the participants used the doormat every day. On the other hand, the life style may differ so that urban participants enter through their front door (and thus presumably use the mat) fewer times than people who live in the countryside. Possible differences in living style between urban and rural participants do not change the findings on the differences in microbes brought indoors, but they can evidently be taken into account in future studies to better explain how environmental doormat microbes enter the house.

Another possible confounding factor for our findings about the association of coverage of built area with the diversity and richness of individual bacterial taxa could be the choice of sampling method. We chose to calculate the diversity and richness of overall bacterial community and the individual taxa and present the subsequent results by rarefying the sample size to 2,000 sequences but did not do so at the taxa level. This is because the distribution of the sequences belonging to those taxa was uneven and sometimes rare across samples. For example, some doormat samples contained fewer than 10 Firmicutes sequences whereas others contained more than 2000. Rarefying the sample size at the individual taxa level would have resulted in excluding those urban doormat samples that had the fewest sequences, thereby increasing the probability of type II error i.e., accepting a false null hypothesis. However, to quench our curiosity, we still calculated the diversity indices and richness for the individual taxa by rarefying the sample size. We found that the association between diversity and richness of Proteobacteria (and its classes) and Bacteroidetes, the two most abundant phyla, and the percentage of built environment within 200 m remained unchanged. The richness of Firmicutes still had an inverse association with percentage of built area while its diversity became independent of built area. The association between the diversity and richness of Actinobacteria and the percentage of built area disappeared. Therefore, we concluded that rarefying the sample size of individual taxa minimally affected our results.

Our finding that the immediate vicinity surrounding the homes of aging urban dwellers is important in determining indoor exposure to diverse environmental microbes should have implications on urban planning. Our study points out the importance of animals in determining Gammaproteobacterial community; if urban planning allows pets to satisfy their natural tendency to interact with soil and vegetation, the pets may aid in maintaining diverse microbiota indoors, as speculated earlier by Barberán et al. (2015). Regardless of the efforts concentrating on urban planning, our view is that novel solutions to introduce rural microbiota indoors are needed, for instance by developing innovative consumer products that harbor forest biodiversity (Puhakka et al., 2017). We envision that if the innovative solutions and urban planning act jointly, urban dwellers will have an option to be in contact with environmental microbial diversity that has been associated with a reduced probability of immune-mediated and other non-communicable diseases.

## Conclusion

We demonstrated that increased level of urbanization reduces transfer of litter and environmental microbiota to homes of aging people. The reduced transfer is ubiquitous, excluding only taxa commonly associated with gut microflora and potential pathogens. In future, an increasing number of people is expected to face the adverse effects of reduced exposure to environmental microbiota and at the same time increased exposure to potentially pathogenic bacteria. We call for research and actions to facilitate efficient transfer of non-pathogenic environmental microbiota indoors in urban, densely built areas.

## Author contributions

AP, MG, OL, HH, RP, and AS: designed the experiments; AP, MG, and AS: designed the manuscript; AP, NS, MG, RP, HV, and MR: performed the experiments; AP, MG, AJ, and AS: analyzed the data; AP, MG, MR, OL, HH, RP, NN, JR, and AS: wrote the manuscript. All authors reviewed the manuscript and gave final approval for publishing the article.

### Conflict of interest statement

The authors declare that the research was conducted in the absence of any commercial or financial relationships that could be construed as a potential conflict of interest.
