# Supplementary material for: Urbanization Reduces Transfer of Diverse Environmental Microbiota Indoors
Source: Front Microbiol. 2018 Feb 5;9:84. doi: 10.3389/fmicb.2018.00084 (PMC5808279; doi:10.3389/fmicb.2018.00084)
Supplement: Supplementary file 1 [file Table1.DOCX]

**Supplementary Table S1.** Mean and standard deviation of the percentage abundance of detected phyla in doormat samples. Due to large standard deviations (SD) between samples, percentage abundances were similar in rural and urban samples.

|  | **Rural** |  | **Urban** |  | **Combined** | |
| --- | --- | --- | --- | --- | --- | --- |
| **Phyla** | Mean | SD | Mean | SD | Mean | SD |
| **Acidobacteria** | 5.13 | 1.74 | 4.62 | 1.98 | 4.91 | 1.84 |
| **Actinobacteria** | 19.36 | 4.15 | 15.09 | 5.31 | 17.57 | 5.09 |
| **Aquificae** | 0.00 | 0.01 | 0.01 | 0.02 | 0.01 | 0.02 |
| **Armatimonadetes** | 0.77 | 0.49 | 0.85 | 0.46 | 0.80 | 0.47 |
| **Bacteria_unclassified** | 14.65 | 5.09 | 14.36 | 7.33 | 14.53 | 6.06 |
| **Bacteroidetes** | 23.79 | 4.67 | 23.93 | 8.91 | 23.85 | 6.70 |
| **candidate_division_WPS-1** | 0.06 | 0.06 | 0.05 | 0.07 | 0.06 | 0.06 |
| **candidate_division_WPS-2** | 0.28 | 0.15 | 0.29 | 0.19 | 0.28 | 0.17 |
| **Candidatus_Saccharibacteria** | 0.01 | 0.02 | 0.00 | 0.00 | 0.01 | 0.02 |
| **Chlamydiae** | 0.19 | 0.56 | 0.07 | 0.10 | 0.14 | 0.43 |
| **Chloroflexi** | 1.28 | 1.09 | 0.90 | 0.92 | 1.12 | 1.03 |
| **Deferribacteres** | 0.01 | 0.05 | 0.00 | 0.01 | 0.01 | 0.04 |
| **Deinococcus-Thermus** | 0.90 | 0.43 | 0.65 | 0.55 | 0.80 | 0.50 |
| **Elusimicrobia** | 0.00 | 0.01 | 0.00 | 0.00 | 0.00 | 0.01 |
| **Fibrobacteres** | 0.01 | 0.06 | 0.00 | 0.00 | 0.01 | 0.05 |
| **Firmicutes** | 8.09 | 7.61 | 10.79 | 12.05 | 9.23 | 9.70 |
| **Fusobacteria** | 0.33 | 0.73 | 0.70 | 2.06 | 0.48 | 1.44 |
| **Gemmatimonadetes** | 0.56 | 0.30 | 0.45 | 0.32 | 0.51 | 0.31 |
| **Lentisphaerae** | 0.01 | 0.03 | 0.00 | 0.00 | 0.00 | 0.02 |
| **Nitrospirae** | 0.05 | 0.05 | 0.02 | 0.04 | 0.04 | 0.05 |
| **Parcubacteria** | 0.02 | 0.03 | 0.02 | 0.05 | 0.02 | 0.04 |
| **Planctomycetes** | 1.15 | 0.44 | 1.16 | 0.81 | 1.16 | 0.61 |
| **Proteobacteria** | 21.30 | 4.60 | 23.95 | 7.74 | 22.42 | 6.19 |
| **Spirochaetes** | 0.04 | 0.09 | 0.00 | 0.01 | 0.02 | 0.07 |
| **Tenericutes** | 0.13 | 0.21 | 0.12 | 0.31 | 0.13 | 0.25 |
| **Verrucomicrobia** | 1.88 | 0.96 | 1.96 | 1.38 | 1.91 | 1.15 |
