# Supplementary material for: Urbanization Reduces Transfer of Diverse Environmental Microbiota Indoors
Source: Front Microbiol. 2018 Feb 5;9:84. doi: 10.3389/fmicb.2018.00084 (PMC5808279; doi:10.3389/fmicb.2018.00084)
Supplement: Supplementary file 2 [file Table2.DOCX]

Supplementary Table S2. The stepwise linear regression analysis statistics of the diversity (Shannon’s index) and richness of the whole bacterial community and all the major phyla against the land use (explanatory variables) revealing the AIC values for all the tested models. (Shannon.all.OTU = Shannon’s diversity index of all bacterial community at the OUT level, shannon.proteo = Shannon’s diversity index for Proteobacteria, shannon.alphaproteo = Shannon’s diversity index for Alphaproteobacteria, shannon.betaproteo = Shannon’s diversity index for Betaproteobacteria, shannon.gammaproteo = Shannon’s diversity index for Gammaproteobacteria, shannon.actino = Shannon’s diversity index for Actinobacteria, shannon.bact = Shannon’s diversity index for Bacteroidetes, shannon.firmi = Shannon’s diversity index for Firmicutes, (rich.all.OTU = richness of all bacterial community at the OTU level, rich.proteo = richness of Proteobacteria, rich.alphaproteo = richness for Alphaproteobacteria, rich.betaproteo = richness of Betaproteobacteria, rich.gammaproteo = richness of Gammaproteobacteria, rich.actino = richness of Actinobacteria, rich.bact = richness of Bacteroidetes, rich.firmi = richness of Firmicutes,perc.built = percentage of built area within 200 m of the study sites, perc.forest = percentage of forest within 200 m of the study sites, perc.trans = percentage of transitional area within 200 m of the study sites).

**Whole bacterial community at the OTU level (Shannon’s diversity index)**

Start: AIC=-66.84

shannon.all.OTU ~ perc.built + perc.forest + perc.trans

Df Sum of Sq RSS AIC

- perc.trans 1 0.0038 11.196 -68.822

- perc.forest 1 0.0734 11.266 -68.512

<none> 11.193 -66.839

- perc.built 1 3.9371 15.130 -53.769

Step: AIC=-68.82

shannon.all.OTU ~ perc.built + perc.forest

Df Sum of Sq RSS AIC

- perc.forest 1 0.0734 11.270 -70.495

<none> 11.196 -68.822

+ perc.trans 1 0.0038 11.193 -66.839

- perc.built 1 3.9341 15.130 -55.766

Step: AIC=-70.5

shannon.all.OTU ~ perc.built

Df Sum of Sq RSS AIC

<none> 11.270 -70.495

+ perc.forest 1 0.0734 11.196 -68.822

+ perc.trans 1 0.0039 11.266 -68.512

- perc.built 1 5.0620 16.332 -53.946

> stepAll$anova

Stepwise Model Path

Analysis of Deviance Table

Initial Model:

shannon.all.OTU ~ perc.built + perc.forest + perc.trans

Final Model:

shannon.all.OTU ~ perc.built

Step Df Deviance Resid. Df Resid. Dev AIC

1 46 11.19253 -66.83884

2 - perc.trans 1 0.003835661 47 11.19636 -68.82170

3 - perc.forest 1 0.073392918 48 11.26975 -70.49502

Residuals:

Min 1Q Median 3Q Max

-1.36680 -0.18642 0.05396 0.30994 0.94318

Coefficients:

Estimate Std. Error t value Pr(>|t|)

(Intercept) 6.167754 0.218804 28.189 < 2e-16 ***

perc.built -0.010786 0.002681 -4.023 0.000212 ***

perc.forest 0.002074 0.003777 0.549 0.585566

perc.trans 0.001724 0.013735 0.126 0.900631

---

Signif. codes: 0 ‘***’ 0.001 ‘**’ 0.01 ‘*’ 0.05 ‘.’ 0.1 ‘ ’ 1

Residual standard error: 0.4933 on 46 degrees of freedom

Multiple R-squared: 0.3147, Adjusted R-squared: 0.27

F-statistic: 7.041 on 3 and 46 DF, p-value: 0.0005412

Residuals:

Min 1Q Median 3Q Max

-1.38065 -0.20830 0.05341 0.30515 0.92124

Coefficients:

Estimate Std. Error t value Pr(>|t|)

(Intercept) 6.259989 0.109445 57.197 < 2e-16 ***

perc.built -0.011333 0.002441 -4.643 2.69e-05 ***

---

Signif. codes: 0 ‘***’ 0.001 ‘**’ 0.01 ‘*’ 0.05 ‘.’ 0.1 ‘ ’ 1

Residual standard error: 0.4845 on 48 degrees of freedom

Multiple R-squared: 0.3099, Adjusted R-squared: 0.2956

F-statistic: 21.56 on 1 and 48 DF, p-value: 2.685e-05

**Proteobacteria (Shannon’s diversity index)**

Start: AIC=-90.47

shannon.proteo ~ perc.built + perc.forest + perc.trans

Df Sum of Sq RSS AIC

- perc.trans 1 0.1193 7.0968 -91.619

<none> 6.9775 -90.467

- perc.forest 1 0.2883 7.2658 -90.442

- perc.built 1 3.9380 10.9155 -70.092

Step: AIC=-91.62

shannon.proteo ~ perc.built + perc.forest

Df Sum of Sq RSS AIC

- perc.forest 1 0.2885 7.3853 -91.626

<none> 7.0968 -91.619

+ perc.trans 1 0.1193 6.9775 -90.467

- perc.built 1 3.9171 11.0139 -71.643

Step: AIC=-91.63

shannon.proteo ~ perc.built

Df Sum of Sq RSS AIC

<none> 7.3853 -91.626

+ perc.forest 1 0.2885 7.0968 -91.619

+ perc.trans 1 0.1195 7.2658 -90.442

- perc.built 1 5.5372 12.9226 -65.652

> stepP$anova

Stepwise Model Path

Analysis of Deviance Table

Initial Model:

shannon.proteo ~ perc.built + perc.forest + perc.trans

Final Model:

shannon.proteo ~ perc.built

Step Df Deviance Resid. Df Resid. Dev AIC

1 46 6.977513 -90.46652

2 - perc.trans 1 0.1192819 47 7.096795 -91.61898

3 - perc.forest 1 0.2885387 48 7.385334 -91.62634

Call:

lm(formula = shannon.proteo ~ perc.built)

Residuals:

Min 1Q Median 3Q Max

-1.23679 -0.22850 0.03403 0.19849 0.73752

Coefficients:

Estimate Std. Error t value Pr(>|t|)

(Intercept) 5.046639 0.088598 56.961 < 2e-16 ***

perc.built -0.011854 0.001976 -5.999 2.52e-07 ***

---

Signif. codes: 0 ‘***’ 0.001 ‘**’ 0.01 ‘*’ 0.05 ‘.’ 0.1 ‘ ’ 1

Residual standard error: 0.3923 on 48 degrees of freedom

Multiple R-squared: 0.4285, Adjusted R-squared: 0.4166

F-statistic: 35.99 on 1 and 48 DF, p-value: 2.516e-07

> summary(reg.shannon.proteo)

Call:

lm(formula = shannon.proteo ~ perc.built + perc.forest + perc.trans)

Residuals:

Min 1Q Median 3Q Max

-1.13848 -0.21747 0.05175 0.18804 0.78444

Coefficients:

Estimate Std. Error t value Pr(>|t|)

(Intercept) 4.806364 0.172759 27.821 < 2e-16 ***

perc.built -0.010787 0.002117 -5.095 6.38e-06 ***

perc.forest 0.004111 0.002982 1.379 0.175

perc.trans 0.009617 0.010844 0.887 0.380

---

Signif. codes: 0 ‘***’ 0.001 ‘**’ 0.01 ‘*’ 0.05 ‘.’ 0.1 ‘ ’ 1

Residual standard error: 0.3895 on 46 degrees of freedom

Multiple R-squared: 0.4601, Adjusted R-squared: 0.4248

F-statistic: 13.06 on 3 and 46 DF, p-value: 2.667e-06

**Alphaproteobacteria (Shannon’s diversity index)**

Start: AIC=-106.26

shannon.alphaproteo ~ perc.built + perc.forest + perc.trans

Df Sum of Sq RSS AIC

- perc.trans 1 0.12451 5.2119 -107.05

<none> 5.0874 -106.26

- perc.forest 1 0.21780 5.3052 -106.17

- perc.built 1 0.74100 5.8284 -101.46

Step: AIC=-107.05

shannon.alphaproteo ~ perc.built + perc.forest

Df Sum of Sq RSS AIC

<none> 5.2119 -107.05

- perc.forest 1 0.21799 5.4299 -107.00

+ perc.trans 1 0.12451 5.0874 -106.26

- perc.built 1 0.73152 5.9434 -102.49

> stepA$anova

Stepwise Model Path

Analysis of Deviance Table

Initial Model:

shannon.alphaproteo ~ perc.built + perc.forest + perc.trans

Final Model:

shannon.alphaproteo ~ perc.built + perc.forest

Step Df Deviance Resid. Df Resid. Dev AIC

1 46 5.087412 -106.2627

2 - perc.trans 1 0.1245092 47 5.211921 -107.0537

> summary(reg.shannon.alpha)

Call:

lm(formula = shannon.alphaproteo ~ perc.built + perc.forest +

perc.trans)

Residuals:

Min 1Q Median 3Q Max

-0.93819 -0.12406 -0.01455 0.21072 0.59493

Coefficients:

Estimate Std. Error t value Pr(>|t|)

(Intercept) 4.196660 0.147516 28.449 <2e-16 ***

perc.built -0.004679 0.001808 -2.588 0.0129 *

perc.forest 0.003573 0.002546 1.403 0.1672

perc.trans 0.009825 0.009260 1.061 0.2942

---

Signif. codes: 0 ‘***’ 0.001 ‘**’ 0.01 ‘*’ 0.05 ‘.’ 0.1 ‘ ’ 1

Residual standard error: 0.3326 on 46 degrees of freedom

Multiple R-squared: 0.2369, Adjusted R-squared: 0.1871

F-statistic: 4.759 on 3 and 46 DF, p-value: 0.005676

> summary(stepA)

Call:

lm(formula = shannon.alphaproteo ~ perc.built + perc.forest)

Residuals:

Min 1Q Median 3Q Max

-0.96175 -0.12528 0.02546 0.20936 0.58951

Coefficients:

Estimate Std. Error t value Pr(>|t|)

(Intercept) 4.287651 0.120189 35.674 <2e-16 ***

perc.built -0.004649 0.001810 -2.568 0.0135 *

perc.forest 0.003575 0.002550 1.402 0.1675

---

Signif. codes: 0 ‘***’ 0.001 ‘**’ 0.01 ‘*’ 0.05 ‘.’ 0.1 ‘ ’ 1

Residual standard error: 0.333 on 47 degrees of freedom

Multiple R-squared: 0.2182, Adjusted R-squared: 0.1849

F-statistic: 6.559 on 2 and 47 DF, p-value: 0.003075

**Betaproteobacteria (Shannon’s diversity index)**

Start: AIC=-91.1

shannon.betaproteo ~ perc.built + perc.forest + perc.trans

Df Sum of Sq RSS AIC

- perc.forest 1 0.06854 6.9576 -92.609

<none> 6.8891 -91.104

- perc.trans 1 0.60202 7.4911 -88.915

- perc.built 1 1.09711 7.9862 -85.715

Step: AIC=-92.61

shannon.betaproteo ~ perc.built + perc.trans

Df Sum of Sq RSS AIC

<none> 6.9576 -92.609

+ perc.forest 1 0.06854 6.8891 -91.104

- perc.trans 1 0.60178 7.5594 -90.461

- perc.built 1 1.52819 8.4858 -84.681

> stepB$anova

Stepwise Model Path

Analysis of Deviance Table

Initial Model:

shannon.betaproteo ~ perc.built + perc.forest + perc.trans

Final Model:

shannon.betaproteo ~ perc.built + perc.trans

Step Df Deviance Resid. Df Resid. Dev AIC

1 46 6.889110 -91.10405

2 - perc.forest 1 0.06853528 47 6.957646 -92.60909

> summary(reg.shannon.beta)

Call:

lm(formula = shannon.betaproteo ~ perc.built + perc.forest +

perc.trans)

Residuals:

Min 1Q Median 3Q Max

-1.02590 -0.17815 0.07273 0.22917 0.72994

Coefficients:

Estimate Std. Error t value Pr(>|t|)

(Intercept) 3.066202 0.171661 17.862 < 2e-16 ***

perc.built -0.005694 0.002104 -2.707 0.00951 **

perc.forest 0.002004 0.002963 0.676 0.50212

perc.trans -0.021604 0.010776 -2.005 0.05087 .

---

Signif. codes: 0 ‘***’ 0.001 ‘**’ 0.01 ‘*’ 0.05 ‘.’ 0.1 ‘ ’ 1

Residual standard error: 0.387 on 46 degrees of freedom

Multiple R-squared: 0.2447, Adjusted R-squared: 0.1954

F-statistic: 4.967 on 3 and 46 DF, p-value: 0.004542

> summary(stepB)

Call:

lm(formula = shannon.betaproteo ~ perc.built + perc.trans)

Residuals:

Min 1Q Median 3Q Max

-1.05879 -0.14501 0.04317 0.21916 0.75080

Coefficients:

Estimate Std. Error t value Pr(>|t|)

(Intercept) 3.139858 0.131943 23.797 < 2e-16 ***

perc.built -0.006228 0.001938 -3.213 0.00237 **

perc.trans -0.021600 0.010713 -2.016 0.04951 *

---

Signif. codes: 0 ‘***’ 0.001 ‘**’ 0.01 ‘*’ 0.05 ‘.’ 0.1 ‘ ’ 1

Residual standard error: 0.3848 on 47 degrees of freedom

Multiple R-squared: 0.2372, Adjusted R-squared: 0.2047

F-statistic: 7.306 on 2 and 47 DF, p-value: 0.001726

**Gammaproteobacteria (Shannon’s diversity index)**

Start: AIC=-63.07

shannon.gammaproteo ~ perc.built + perc.forest + perc.trans

Df Sum of Sq RSS AIC

- perc.trans 1 0.0066 12.074 -65.047

- perc.forest 1 0.2648 12.332 -63.989

<none> 12.068 -63.074

- perc.built 1 5.8177 17.885 -45.402

Step: AIC=-65.05

shannon.gammaproteo ~ perc.built + perc.forest

Df Sum of Sq RSS AIC

- perc.forest 1 0.2648 12.339 -65.963

<none> 12.074 -65.047

+ perc.trans 1 0.0066 12.068 -63.074

- perc.built 1 5.8254 17.900 -47.362

Step: AIC=-65.96

shannon.gammaproteo ~ perc.built

Df Sum of Sq RSS AIC

<none> 12.339 -65.963

+ perc.forest 1 0.2648 12.074 -65.047

+ perc.trans 1 0.0065 12.332 -63.989

- perc.built 1 7.9113 20.250 -43.193

> stepG$anova

Stepwise Model Path

Analysis of Deviance Table

Initial Model:

shannon.gammaproteo ~ perc.built + perc.forest + perc.trans

Final Model:

shannon.gammaproteo ~ perc.built

Step Df Deviance Resid. Df Resid. Dev AIC

1 46 12.06771 -63.07448

2 - perc.trans 1 0.006575916 47 12.07429 -65.04724

3 - perc.forest 1 0.264772148 48 12.33906 -65.96266

Residuals:

Min 1Q Median 3Q Max

-1.66904 -0.25236 -0.07139 0.35691 1.16486

Coefficients:

Estimate Std. Error t value Pr(>|t|)

(Intercept) 3.123555 0.227197 13.748 < 2e-16 ***

perc.built -0.013111 0.002784 -4.709 2.32e-05 ***

perc.forest 0.003940 0.003922 1.005 0.320

perc.trans -0.002258 0.014262 -0.158 0.875

---

Signif. codes: 0 ‘***’ 0.001 ‘**’ 0.01 ‘*’ 0.05 ‘.’ 0.1 ‘ ’ 1

Residual standard error: 0.5122 on 46 degrees of freedom

Multiple R-squared: 0.4041, Adjusted R-squared: 0.3652

F-statistic: 10.4 on 3 and 46 DF, p-value: 2.431e-05

Call:

lm(formula = shannon.gammaproteo ~ perc.built)

Residuals:

Min 1Q Median 3Q Max

-1.68393 -0.27647 -0.01527 0.27558 1.20794

Coefficients:

Estimate Std. Error t value Pr(>|t|)

(Intercept) 3.247497 0.114520 28.358 < 2e-16 ***

perc.built -0.014169 0.002554 -5.548 1.22e-06 ***

---

Signif. codes: 0 ‘***’ 0.001 ‘**’ 0.01 ‘*’ 0.05 ‘.’ 0.1 ‘ ’ 1

Residual standard error: 0.507 on 48 degrees of freedom

Multiple R-squared: 0.3907, Adjusted R-squared: 0.378

F-statistic: 30.78 on 1 and 48 DF, p-value: 1.221e-06

**Actinobacteria (Shannon’s diversity index)**

Start: AIC=-91.33

shannon.Actino ~ perc.built + perc.forest + perc.trans

Df Sum of Sq RSS AIC

- perc.forest 1 0.08201 6.9403 -92.734

- perc.trans 1 0.09974 6.9580 -92.607

<none> 6.8583 -91.328

- perc.built 1 0.66949 7.5278 -88.671

Step: AIC=-92.73

shannon.Actino ~ perc.built + perc.trans

Df Sum of Sq RSS AIC

- perc.trans 1 0.09963 7.0399 -94.021

<none> 6.9403 -92.734

+ perc.forest 1 0.08201 6.8583 -91.328

- perc.built 1 0.99771 7.9380 -88.018

Step: AIC=-94.02

shannon.Actino ~ perc.built

Df Sum of Sq RSS AIC

<none> 7.0399 -94.021

+ perc.trans 1 0.09963 6.9403 -92.734

+ perc.forest 1 0.08190 6.9580 -92.607

- perc.built 1 1.00874 8.0486 -89.326

> stepAc$anova

Stepwise Model Path

Analysis of Deviance Table

Initial Model:

shannon.Actino ~ perc.built + perc.forest + perc.trans

Final Model:

shannon.Actino ~ perc.built

Step Df Deviance Resid. Df Resid. Dev AIC

1 46 6.858267 -91.32841

2 - perc.forest 1 0.08201033 47 6.940277 -92.73407

3 - perc.trans 1 0.09962783 48 7.039905 -94.02142

Residuals:

Min 1Q Median 3Q Max

-1.40864 -0.22557 0.04749 0.25834 0.57226

Coefficients:

Estimate Std. Error t value Pr(>|t|)

(Intercept) 4.342210 0.171276 25.352 <2e-16 ***

perc.built -0.004448 0.002099 -2.119 0.0395 *

perc.forest 0.002193 0.002956 0.742 0.4621

perc.trans -0.008794 0.010751 -0.818 0.4176

---

Signif. codes: 0 ‘***’ 0.001 ‘**’ 0.01 ‘*’ 0.05 ‘.’ 0.1 ‘ ’ 1

Residual standard error: 0.3861 on 46 degrees of freedom

Multiple R-squared: 0.1479, Adjusted R-squared: 0.09233

F-statistic: 2.661 on 3 and 46 DF, p-value: 0.05911

Call:

lm(formula = shannon.Actino ~ perc.built)

Residuals:

Min 1Q Median 3Q Max

-1.38550 -0.19874 0.01876 0.27402 0.55494

Coefficients:

Estimate Std. Error t value Pr(>|t|)

(Intercept) 4.341336 0.086501 50.188 <2e-16 ***

perc.built -0.005059 0.001929 -2.623 0.0117 *

Signif. codes: 0 ‘***’ 0.001 ‘**’ 0.01 ‘*’ 0.05 ‘.’ 0.1 ‘ ’ 1

Residual standard error: 0.383 on 48 degrees of freedom

Multiple R-squared: 0.1253, Adjusted R-squared: 0.1071

F-statistic: 6.878 on 1 and 48 DF, p-value: 0.01166

**Bacteroidetes (Shannon’s diversity index)**

Start: AIC=-52.14

shannon.Bact ~ perc.built + perc.forest + perc.trans

Df Sum of Sq RSS AIC

- perc.trans 1 0.00008 15.019 -54.135

- perc.forest 1 0.01257 15.031 -54.094

<none> 15.019 -52.136

- perc.built 1 2.49147 17.510 -46.461

Step: AIC=-54.14

shannon.Bact ~ perc.built + perc.forest

Df Sum of Sq RSS AIC

- perc.forest 1 0.01257 15.031 -56.094

<none> 15.019 -54.135

+ perc.trans 1 0.00008 15.019 -52.136

- perc.built 1 2.49165 17.511 -48.461

Step: AIC=-56.09

shannon.Bact ~ perc.built

Df Sum of Sq RSS AIC

<none> 15.031 -56.094

+ perc.forest 1 0.01257 15.019 -54.135

+ perc.trans 1 0.00009 15.031 -54.094

- perc.built 1 3.05753 18.089 -48.836

> stepBac$anova

Stepwise Model Path

Analysis of Deviance Table

Initial Model:

shannon.Bact ~ perc.built + perc.forest + perc.trans

Final Model:

shannon.Bact ~ perc.built

Step Df Deviance Resid. Df Resid. Dev AIC

1 46 15.01890 -52.13569

2 - perc.trans 1 8.376064e-05 47 15.01898 -54.13541

3 - perc.forest 1 1.256664e-02 48 15.03155 -56.09360

Residuals:

Min 1Q Median 3Q Max

-1.83937 -0.11386 0.08144 0.33630 0.76525

Coefficients:

Estimate Std. Error t value Pr(>|t|)

(Intercept) 4.4934910 0.2534600 17.729 < 2e-16 ***

perc.built -0.0085802 0.0031060 -2.762 0.00822 **

perc.forest 0.0008583 0.0043750 0.196 0.84534

perc.trans 0.0002548 0.0159103 0.016 0.98729

---

Signif. codes: 0 ‘***’ 0.001 ‘**’ 0.01 ‘*’ 0.05 ‘.’ 0.1 ‘ ’ 1

Residual standard error: 0.5714 on 46 degrees of freedom

Multiple R-squared: 0.1697, Adjusted R-squared: 0.1156

F-statistic: 3.134 on 3 and 46 DF, p-value: 0.03438

Call:

lm(formula = shannon.Bact ~ perc.built)

Residuals:

Min 1Q Median 3Q Max

-1.81882 -0.12184 0.08136 0.34572 0.77395

Coefficients:

Estimate Std. Error t value Pr(>|t|)

(Intercept) 4.527408 0.126398 35.819 < 2e-16 ***

perc.built -0.008808 0.002819 -3.125 0.00302 **

---

Signif. codes: 0 ‘***’ 0.001 ‘**’ 0.01 ‘*’ 0.05 ‘.’ 0.1 ‘ ’ 1

Residual standard error: 0.5596 on 48 degrees of freedom

Multiple R-squared: 0.169, Adjusted R-squared: 0.1517

F-statistic: 9.764 on 1 and 48 DF, p-value: 0.003017

**Firmicutes (Shannon’s diversity index)**

Start: AIC=-19.86

shannon.Firmi ~ perc.built + perc.forest + perc.trans

Df Sum of Sq RSS AIC

- perc.forest 1 0.00186 28.640 -21.861

- perc.trans 1 0.41021 29.048 -21.153

<none> 28.638 -19.864

- perc.built 1 2.21359 30.852 -18.142

Step: AIC=-21.86

shannon.Firmi ~ perc.built + perc.trans

Df Sum of Sq RSS AIC

- perc.trans 1 0.41025 29.050 -23.150

<none> 28.640 -21.861

+ perc.forest 1 0.00186 28.638 -19.864

- perc.built 1 2.52110 31.161 -19.643

Step: AIC=-23.15

shannon.Firmi ~ perc.built

Df Sum of Sq RSS AIC

<none> 29.050 -23.150

+ perc.trans 1 0.41025 28.640 -21.861

+ perc.forest 1 0.00190 29.048 -21.153

- perc.built 1 2.55650 31.607 -20.933

> stepFir$anova

Stepwise Model Path

Analysis of Deviance Table

Initial Model:

shannon.Firmi ~ perc.built + perc.forest + perc.trans

Final Model:

shannon.Firmi ~ perc.built

Step Df Deviance Resid. Df Resid. Dev AIC

1 46 28.63797 -19.86447

2 - perc.forest 1 0.001863548 47 28.63984 -21.86122

3 - perc.trans 1 0.410247666 48 29.05009 -23.15008

Residuals:

Min 1Q Median 3Q Max

-1.9135 -0.4610 0.2241 0.6573 1.1435

Coefficients:

Estimate Std. Error t value Pr(>|t|)

(Intercept) 3.4674264 0.3499948 9.907 5.47e-13 ***

perc.built -0.0080875 0.0042890 -1.886 0.0657 .

perc.forest -0.0003305 0.0060413 -0.055 0.9566

perc.trans -0.0178338 0.0219700 -0.812 0.4211

---

Signif. codes: 0 ‘***’ 0.001 ‘**’ 0.01 ‘*’ 0.05 ‘.’ 0.1 ‘ ’ 1

Residual standard error: 0.789 on 46 degrees of freedom

Multiple R-squared: 0.09392, Adjusted R-squared: 0.03483

F-statistic: 1.589 on 3 and 46 DF, p-value: 0.2048

Call:

lm(formula = shannon.Firmi ~ perc.built)

Residuals:

Min 1Q Median 3Q Max

-1.9906 -0.5148 0.1878 0.6413 1.1160

Coefficients:

Estimate Std. Error t value Pr(>|t|)

(Intercept) 3.290006 0.175717 18.723 <2e-16 ***

perc.built -0.008054 0.003919 -2.055 0.0453 *

---

Signif. codes: 0 ‘***’ 0.001 ‘**’ 0.01 ‘*’ 0.05 ‘.’ 0.1 ‘ ’ 1

Residual standard error: 0.778 on 48 degrees of freedom

Multiple R-squared: 0.08089, Adjusted R-squared: 0.06174

F-statistic: 4.224 on 1 and 48 DF, p-value: 0.04532

**Whole bacterial community (richness)**

Start: AIC=485.94

rich.all.OTU ~ perc.built + perc.forest + perc.trans

Df Sum of Sq RSS AIC

- perc.trans 1 410 708817 483.97

- perc.forest 1 13440 721848 484.88

<none> 708408 485.94

- perc.built 1 198457 906865 496.29

Step: AIC=483.97

rich.all.OTU ~ perc.built + perc.forest

Df Sum of Sq RSS AIC

- perc.forest 1 13443 722261 482.91

<none> 708817 483.97

+ perc.trans 1 410 708408 485.94

- perc.built 1 198220 907037 494.30

Step: AIC=482.91

rich.all.OTU ~ perc.built

Df Sum of Sq RSS AIC

<none> 722261 482.91

+ perc.forest 1 13443 708817 483.97

+ perc.trans 1 412 721848 484.88

- perc.built 1 278110 1000370 497.19

> stepAllR$anova

Stepwise Model Path

Analysis of Deviance Table

Initial Model:

rich.all.OTU ~ perc.built + perc.forest + perc.trans

Final Model:

rich.all.OTU ~ perc.built

Step Df Deviance Resid. Df Resid. Dev AIC

1 46 708407.9 485.9376

2 - perc.trans 1 409.5282 47 708817.5 483.9665

3 - perc.forest 1 13443.2831 48 722260.7 482.9059

Residuals:

Min 1Q Median 3Q Max

-339.19 -91.22 12.26 76.84 251.09

Coefficients:

Estimate Std. Error t value Pr(>|t|)

(Intercept) 812.3199 55.0468 14.757 <2e-16 ***

perc.built -2.4216 0.6746 -3.590 0.0008 ***

perc.forest 0.8877 0.9502 0.934 0.3551

perc.trans 0.5635 3.4554 0.163 0.8712

---

Signif. codes: 0 ‘***’ 0.001 ‘**’ 0.01 ‘*’ 0.05 ‘.’ 0.1 ‘ ’ 1

Residual standard error: 124.1 on 46 degrees of freedom

Multiple R-squared: 0.2919, Adjusted R-squared: 0.2457

F-statistic: 6.319 on 3 and 46 DF, p-value: 0.001112

Call:

lm(formula = rich.all.OTU ~ perc.built)

Residuals:

Min 1Q Median 3Q Max

-344.99 -103.54 19.44 88.84 247.27

Coefficients:

Estimate Std. Error t value Pr(>|t|)

(Intercept) 850.1779 27.7068 30.685 < 2e-16 ***

perc.built -2.6565 0.6179 -4.299 8.35e-05 ***

---

Signif. codes: 0 ‘***’ 0.001 ‘**’ 0.01 ‘*’ 0.05 ‘.’ 0.1 ‘ ’ 1

Residual standard error: 122.7 on 48 degrees of freedom

Multiple R-squared: 0.278, Adjusted R-squared: 0.263

F-statistic: 18.48 on 1 and 48 DF, p-value: 8.348e-05

**Proteobacteria (richness)**

Start: AIC=356.25

rich.proteo ~ perc.built + perc.forest + perc.trans

Df Sum of Sq RSS AIC

- perc.trans 1 5.7 52950 354.25

- perc.forest 1 1343.1 54287 355.50

<none> 52944 356.25

- perc.built 1 7265.9 60210 360.68

Step: AIC=354.25

rich.proteo ~ perc.built + perc.forest

Df Sum of Sq RSS AIC

- perc.forest 1 1343.2 54293 353.51

<none> 52950 354.25

+ perc.trans 1 5.7 52944 356.25

- perc.built 1 7261.3 60211 358.68

Step: AIC=353.51

rich.proteo ~ perc.built

Df Sum of Sq RSS AIC

<none> 54293 353.51

+ perc.forest 1 1343.2 52950 354.25

+ perc.trans 1 5.8 54287 355.50

- perc.built 1 11404.9 65698 361.04

> stepPR$anova

Stepwise Model Path

Analysis of Deviance Table

Initial Model:

rich.proteo ~ perc.built + perc.forest + perc.trans

Final Model:

rich.proteo ~ perc.built

Step Df Deviance Resid. Df Resid. Dev AIC

1 46 52944.17 356.2485

2 - perc.trans 1 5.679573 47 52949.85 354.2539

3 - perc.forest 1 1343.241134 48 54293.09 353.5065

Call:

lm(formula = rich.proteo ~ perc.built)

Residuals:

Min 1Q Median 3Q Max

-83.222 -17.593 -2.969 24.462 68.962

Coefficients:

Estimate Std. Error t value Pr(>|t|)

(Intercept) 209.6083 7.5965 27.593 < 2e-16 ***

perc.built -0.5380 0.1694 -3.175 0.00261 **

---

Signif. codes: 0 ‘***’ 0.001 ‘**’ 0.01 ‘*’ 0.05 ‘.’ 0.1 ‘ ’ 1

Residual standard error: 33.63 on 48 degrees of freedom

Multiple R-squared: 0.1736, Adjusted R-squared: 0.1564

F-statistic: 10.08 on 1 and 48 DF, p-value: 0.002614

Call:

lm(formula = rich.proteo ~ perc.built + perc.forest + perc.trans)

Residuals:

Min 1Q Median 3Q Max

-81.472 -16.790 -2.907 24.687 71.839

Coefficients:

Estimate Std. Error t value Pr(>|t|)

(Intercept) 198.67641 15.04872 13.202 <2e-16 ***

perc.built -0.46335 0.18442 -2.513 0.0156 *

perc.forest 0.28061 0.25976 1.080 0.2857

perc.trans 0.06636 0.94464 0.070 0.9443

---

Signif. codes: 0 ‘***’ 0.001 ‘**’ 0.01 ‘*’ 0.05 ‘.’ 0.1 ‘ ’ 1

Residual standard error: 33.93 on 46 degrees of freedom

Multiple R-squared: 0.1941, Adjusted R-squared: 0.1416

F-statistic: 3.694 on 3 and 46 DF, p-value: 0.01829

**Alpharoteobacteria (richness)**

Start: AIC=324.01

rich.alphaproteo ~ perc.built + perc.forest + perc.trans

Df Sum of Sq RSS AIC

- perc.trans 1 91.28 27874 322.17

- perc.forest 1 625.20 28408 323.12

<none> 27782 324.01

- perc.built 1 2175.10 29958 325.78

Step: AIC=322.17

rich.alphaproteo ~ perc.built + perc.forest

Df Sum of Sq RSS AIC

- perc.forest 1 625.49 28499 321.28

<none> 27874 322.17

- perc.built 1 2161.45 30035 323.90

+ perc.trans 1 91.28 27782 324.01

Step: AIC=321.28

rich.alphaproteo ~ perc.built

Df Sum of Sq RSS AIC

<none> 28499 321.28

+ perc.forest 1 625.5 27874 322.17

+ perc.trans 1 91.6 28408 323.12

- perc.built 1 3635.7 32135 325.28

> stepAR$anova

Stepwise Model Path

Analysis of Deviance Table

Initial Model:

rich.alphaproteo ~ perc.built + perc.forest + perc.trans

Final Model:

rich.alphaproteo ~ perc.built

Step Df Deviance Resid. Df Resid. Dev AIC

1 46 27782.38 324.0067

2 - perc.trans 1 91.28381 47 27873.67 322.1707

3 - perc.forest 1 625.48569 48 28499.15 321.2803

Residuals:

Min 1Q Median 3Q Max

-63.14 -16.30 3.31 15.63 43.01

Coefficients:

Estimate Std. Error t value Pr(>|t|)

(Intercept) 109.7952 10.9012 10.072 3.24e-13 ***

perc.built -0.2535 0.1336 -1.898 0.064 .

perc.forest 0.1914 0.1882 1.017 0.314

perc.trans 0.2660 0.6843 0.389 0.699

---

Signif. codes: 0 ‘***’ 0.001 ‘**’ 0.01 ‘*’ 0.05 ‘.’ 0.1 ‘ ’ 1

Residual standard error: 24.58 on 46 degrees of freedom

Multiple R-squared: 0.1354, Adjusted R-squared: 0.07906

F-statistic: 2.402 on 3 and 46 DF, p-value: 0.07974

Call:

lm(formula = rich.alphaproteo ~ perc.built)

Residuals:

Min 1Q Median 3Q Max

-64.070 -16.100 3.283 17.752 39.963

Coefficients:

Estimate Std. Error t value Pr(>|t|)

(Intercept) 119.2994 5.5037 21.676 <2e-16 ***

perc.built -0.3037 0.1227 -2.475 0.0169 *

---

Signif. codes: 0 ‘***’ 0.001 ‘**’ 0.01 ‘*’ 0.05 ‘.’ 0.1 ‘ ’ 1

Residual standard error: 24.37 on 48 degrees of freedom

Multiple R-squared: 0.1131, Adjusted R-squared: 0.09466

F-statistic: 6.124 on 1 and 48 DF, p-value: 0.01693

**Betaproteobacteria (richness)**

Start: AIC=171.26

rich.betaproteo ~ perc.built + perc.forest + perc.trans

Df Sum of Sq RSS AIC

- perc.trans 1 0.039 1309.4 169.26

- perc.forest 1 25.873 1335.2 170.24

<none> 1309.4 171.26

- perc.built 1 119.117 1428.5 173.62

Step: AIC=169.27

rich.betaproteo ~ perc.built + perc.forest

Df Sum of Sq RSS AIC

- perc.forest 1 25.872 1335.3 168.24

<none> 1309.4 169.26

+ perc.trans 1 0.039 1309.4 171.26

- perc.built 1 119.216 1428.6 171.62

Step: AIC=168.24

rich.betaproteo ~ perc.built

Df Sum of Sq RSS AIC

<none> 1335.3 168.24

+ perc.forest 1 25.872 1309.4 169.26

+ perc.trans 1 0.038 1335.2 170.24

- perc.built 1 191.599 1526.9 172.95

> stepBR$anova

Stepwise Model Path

Analysis of Deviance Table

Initial Model:

rich.betaproteo ~ perc.built + perc.forest + perc.trans

Final Model:

rich.betaproteo ~ perc.built

Step Df Deviance Resid. Df Resid. Dev AIC

1 46 1309.370 171.2639

2 - perc.trans 1 0.03928702 47 1309.409 169.2654

3 - perc.forest 1 25.87159001 48 1335.281 168.2437

Residuals:

Min 1Q Median 3Q Max

-11.5833 -3.4122 -0.0179 2.3684 12.6694

Coefficients:

Estimate Std. Error t value Pr(>|t|)

(Intercept) 22.377059 2.366582 9.455 2.34e-12 ***

perc.built -0.059327 0.029001 -2.046 0.0465 *

perc.forest 0.038946 0.040850 0.953 0.3454

perc.trans -0.005519 0.148556 -0.037 0.9705

---

Signif. codes: 0 ‘***’ 0.001 ‘**’ 0.01 ‘*’ 0.05 ‘.’ 0.1 ‘ ’ 1

Residual standard error: 5.335 on 46 degrees of freedom

Multiple R-squared: 0.1425, Adjusted R-squared: 0.08653

F-statistic: 2.547 on 3 and 46 DF, p-value: 0.06743

Call:

lm(formula = rich.betaproteo ~ perc.built)

Residuals:

Min 1Q Median 3Q Max

-12.2343 -3.4187 0.1344 2.1193 12.2784

Coefficients:

Estimate Std. Error t value Pr(>|t|)

(Intercept) 23.75782 1.19131 19.943 <2e-16 ***

perc.built -0.06973 0.02657 -2.624 0.0116 *

---

Signif. codes: 0 ‘***’ 0.001 ‘**’ 0.01 ‘*’ 0.05 ‘.’ 0.1 ‘ ’ 1

Residual standard error: 5.274 on 48 degrees of freedom

Multiple R-squared: 0.1255, Adjusted R-squared: 0.1073

F-statistic: 6.888 on 1 and 48 DF, p-value: 0.01161

**Gammaproteobacteria (richness)**

Call:

lm(formula = rich.gammaproteo ~ perc.built + perc.forest + perc.trans)

Start: AIC=231.05

rich.gammaproteo ~ perc.built + perc.forest + perc.trans

Df Sum of Sq RSS AIC

- perc.forest 1 10.83 4339.2 229.17

- perc.trans 1 169.49 4497.9 230.97

<none> 4328.4 231.05

- perc.built 1 320.55 4648.9 232.62

Step: AIC=229.17

rich.gammaproteo ~ perc.built + perc.trans

Df Sum of Sq RSS AIC

- perc.trans 1 169.43 4508.7 229.09

<none> 4339.2 229.17

+ perc.forest 1 10.83 4328.4 231.05

- perc.built 1 426.46 4765.7 231.86

Step: AIC=229.09

rich.gammaproteo ~ perc.built

Df Sum of Sq RSS AIC

<none> 4508.7 229.09

+ perc.trans 1 169.43 4339.2 229.17

+ perc.forest 1 10.78 4497.9 230.97

- perc.built 1 435.77 4944.4 231.70

> stepGR$anova

Stepwise Model Path

Analysis of Deviance Table

Initial Model:

rich.gammaproteo ~ perc.built + perc.forest + perc.trans

Final Model:

rich.gammaproteo ~ perc.built

Step Df Deviance Resid. Df Resid. Dev AIC

1 46 4328.388 231.0464

2 - perc.forest 1 10.83225 47 4339.221 229.1714

3 - perc.trans 1 169.43387 48 4508.655 229.0866

Residuals:

Min 1Q Median 3Q Max

-17.626 -5.608 -1.015 3.999 23.335

Coefficients:

Estimate Std. Error t value Pr(>|t|)

(Intercept) 39.56926 4.30282 9.196 5.46e-12 ***

perc.built -0.09732 0.05273 -1.846 0.0714 .

perc.forest 0.02520 0.07427 0.339 0.7359

perc.trans -0.36250 0.27010 -1.342 0.1862

---

Signif. codes: 0 ‘***’ 0.001 ‘**’ 0.01 ‘*’ 0.05 ‘.’ 0.1 ‘ ’ 1

Residual standard error: 9.7 on 46 degrees of freedom

Multiple R-squared: 0.1246, Adjusted R-squared: 0.0675

F-statistic: 2.182 on 3 and 46 DF, p-value: 0.1029

Call:

lm(formula = rich.gammaproteo ~ perc.built)

Residuals:

Min 1Q Median 3Q Max

-18.839 -5.788 -1.400 3.570 22.505

Coefficients:

Estimate Std. Error t value Pr(>|t|)

(Intercept) 37.13647 2.18909 16.964 <2e-16 ***

perc.built -0.10515 0.04882 -2.154 0.0363 *

---

Signif. codes: 0 ‘***’ 0.001 ‘**’ 0.01 ‘*’ 0.05 ‘.’ 0.1 ‘ ’ 1

Residual standard error: 9.692 on 48 degrees of freedom

Multiple R-squared: 0.08813, Adjusted R-squared: 0.06914

F-statistic: 4.639 on 1 and 48 DF, p-value: 0.0363

**Bacteroidetes (richness)**

Start: AIC=325.58

rich.Bact ~ perc.built + perc.forest + perc.trans

Df Sum of Sq RSS AIC

- perc.forest 1 1.0 28672 323.58

- perc.trans 1 55.1 28726 323.68

<none> 28671 325.58

- perc.built 1 8471.7 37143 336.52

Step: AIC=323.58

rich.Bact ~ perc.built + perc.trans

Df Sum of Sq RSS AIC

- perc.trans 1 55.1 28727 321.68

<none> 28672 323.58

+ perc.forest 1 1.0 28671 325.58

- perc.built 1 9780.8 38453 336.26

Step: AIC=321.68

rich.Bact ~ perc.built

Df Sum of Sq RSS AIC

<none> 28727 321.68

+ perc.trans 1 55.1 28672 323.58

+ perc.forest 1 1.0 28726 323.68

- perc.built 1 9808.6 38536 334.37

> stepBacR$anova

Stepwise Model Path

Analysis of Deviance Table

Initial Model:

rich.Bact ~ perc.built + perc.forest + perc.trans

Final Model:

rich.Bact ~ perc.built

Step Df Deviance Resid. Df Resid. Dev AIC

1 46 28670.89 325.5807

2 - perc.forest 1 1.029206 47 28671.92 323.5825

3 - perc.trans 1 55.136053 48 28727.06 321.6786

Residuals:

Min 1Q Median 3Q Max

-63.559 -15.358 -2.171 19.451 41.678

Coefficients:

Estimate Std. Error t value Pr(>|t|)

(Intercept) 165.723933 11.074167 14.965 < 2e-16 ***

perc.built -0.500325 0.135709 -3.687 0.000598 ***

perc.forest -0.007768 0.191152 -0.041 0.967762

perc.trans -0.206738 0.695151 -0.297 0.767500

---

Signif. codes: 0 ‘***’ 0.001 ‘**’ 0.01 ‘*’ 0.05 ‘.’ 0.1 ‘ ’ 1

Residual standard error: 24.97 on 46 degrees of freedom

Multiple R-squared: 0.256, Adjusted R-squared: 0.2075

F-statistic: 5.276 on 3 and 46 DF, p-value: 0.003273

Call:

lm(formula = rich.Bact ~ perc.built)

Residuals:

Min 1Q Median 3Q Max

-63.359 -15.406 -1.433 19.093 40.990

Coefficients:

Estimate Std. Error t value Pr(>|t|)

(Intercept) 163.5225 5.5257 29.593 < 2e-16 ***

perc.built -0.4989 0.1232 -4.048 0.000187 ***

Signif. codes: 0 ‘***’ 0.001 ‘**’ 0.01 ‘*’ 0.05 ‘.’ 0.1 ‘ ’ 1

Residual standard error: 24.46 on 48 degrees of freedom

Multiple R-squared: 0.2545, Adjusted R-squared: 0.239

F-statistic: 16.39 on 1 and 48 DF, p-value: 0.000187

**Firmicutes (richness)**

Start: AIC=332.71

rich.Firmi ~ perc.built + perc.forest + perc.trans

Df Sum of Sq RSS AIC

- perc.forest 1 236.64 33303 331.07

<none> 33066 332.71

- perc.built 1 1451.94 34518 332.86

- perc.trans 1 1964.49 35030 333.60

Step: AIC=331.07

rich.Firmi ~ perc.built + perc.trans

Df Sum of Sq RSS AIC

- perc.built 1 1216.64 34519 330.86

<none> 33303 331.07

- perc.trans 1 1965.31 35268 331.94

+ perc.forest 1 236.64 33066 332.71

Step: AIC=330.86

rich.Firmi ~ perc.trans

Df Sum of Sq RSS AIC

<none> 34519 330.86

+ perc.built 1 1216.64 33303 331.07

- perc.trans 1 2018.77 36538 331.70

+ perc.forest 1 1.34 34518 332.86

> stepFirR$anova

Stepwise Model Path

Analysis of Deviance Table

Initial Model:

rich.Firmi ~ perc.built + perc.forest + perc.trans

Final Model:

rich.Firmi ~ perc.trans

Step Df Deviance Resid. Df Resid. Dev AIC

1 46 33065.95 332.7118

2 - perc.forest 1 236.6362 47 33302.58 331.0684

3 - perc.built 1 1216.6434 48 34519.23 330.8624

> summary(reg.rich.Firmi)

Call:

lm(formula = rich.Firmi ~ perc.built + perc.forest + perc.trans)

Residuals:

Min 1Q Median 3Q Max

-51.685 -17.190 -1.182 14.445 71.004

Coefficients:

Estimate Std. Error t value Pr(>|t|)

(Intercept) 68.4439 11.8927 5.755 6.73e-07 ***

perc.built -0.2071 0.1457 -1.421 0.162

perc.forest -0.1178 0.2053 -0.574 0.569

perc.trans -1.2341 0.7465 -1.653 0.105

---

Signif. codes: 0 ‘***’ 0.001 ‘**’ 0.01 ‘*’ 0.05 ‘.’ 0.1 ‘ ’ 1

Residual standard error: 26.81 on 46 degrees of freedom

Multiple R-squared: 0.09503, Adjusted R-squared: 0.03601

F-statistic: 1.61 on 3 and 46 DF, p-value: 0.2

> summary(stepFirR)

Call:

lm(formula = rich.Firmi ~ perc.trans)

Residuals:

Min 1Q Median 3Q Max

-45.523 -18.243 -2.596 11.368 66.052

Coefficients:

Estimate Std. Error t value Pr(>|t|)

(Intercept) 58.1266 7.9605 7.302 2.54e-09 ***

perc.trans -1.2509 0.7466 -1.675 0.1

---

Signif. codes: 0 ‘***’ 0.001 ‘**’ 0.01 ‘*’ 0.05 ‘.’ 0.1 ‘ ’ 1

Residual standard error: 26.82 on 48 degrees of freedom

Multiple R-squared: 0.05525, Adjusted R-squared: 0.03557

F-statistic: 2.807 on 1 and 48 DF, p-value: 0.1003

**Actinobacteria (richness)**

Start: AIC=329.83

rich.Actino ~ perc.built + perc.forest + perc.trans

Df Sum of Sq RSS AIC

- perc.trans 1 119.2 31331 328.02

- perc.forest 1 724.4 31936 328.97

<none> 31212 329.83

- perc.built 1 8810.8 40023 340.26

Step: AIC=328.02

rich.Actino ~ perc.built + perc.forest

Df Sum of Sq RSS AIC

- perc.forest 1 724.1 32055 327.16

<none> 31331 328.02

+ perc.trans 1 119.2 31212 329.83

- perc.built 1 8845.8 40177 338.45

Step: AIC=327.16

rich.Actino ~ perc.built

Df Sum of Sq RSS AIC

<none> 32055 327.16

+ perc.forest 1 724.1 31331 328.02

+ perc.trans 1 118.9 31936 328.97

- perc.built 1 12630.0 44685 341.77

> stepAcR$anova

Stepwise Model Path

Analysis of Deviance Table

Initial Model:

rich.Actino ~ perc.built + perc.forest + perc.trans

Final Model:

rich.Actino ~ perc.built

Step Df Deviance Resid. Df Resid. Dev AIC

1 46 31211.96 329.8267

2 - perc.trans 1 119.2185 47 31331.18 328.0173

3 - perc.forest 1 724.0593 48 32055.24 327.1596

Residuals:

Min 1Q Median 3Q Max

-49.634 -22.802 4.335 20.998 46.351

Coefficients:

Estimate Std. Error t value Pr(>|t|)

(Intercept) 137.3734 11.5545 11.889 1.26e-15 ***

perc.built -0.5102 0.1416 -3.604 0.000768 ***

perc.forest 0.2061 0.1994 1.033 0.306882

perc.trans -0.3040 0.7253 -0.419 0.677043

---

Signif. codes: 0 ‘***’ 0.001 ‘**’ 0.01 ‘*’ 0.05 ‘.’ 0.1 ‘ ’ 1

Residual standard error: 26.05 on 46 degrees of freedom

Multiple R-squared: 0.3015, Adjusted R-squared: 0.256

F-statistic: 6.619 on 3 and 46 DF, p-value: 0.0008226

Call:

lm(formula = rich.Actino ~ perc.built)

Residuals:

Min 1Q Median 3Q Max

-46.145 -22.776 4.739 19.166 43.142

Coefficients:

Estimate Std. Error t value Pr(>|t|)

(Intercept) 142.1327 5.8370 24.350 < 2e-16 ***

perc.built -0.5661 0.1302 -4.349 7.1e-05 ***

---

Signif. codes: 0 ‘***’ 0.001 ‘**’ 0.01 ‘*’ 0.05 ‘.’ 0.1 ‘ ’ 1

Residual standard error: 25.84 on 48 degrees of freedom

Multiple R-squared: 0.2826, Adjusted R-squared: 0.2677

F-statistic: 18.91 on 1 and 48 DF, p-value: 7.1e-05
