# Supplementary material for: Urbanization Reduces Transfer of Diverse Environmental Microbiota Indoors
Source: Front Microbiol. 2018 Feb 5;9:84. doi: 10.3389/fmicb.2018.00084 (PMC5808279; doi:10.3389/fmicb.2018.00084)
Supplement: Supplementary file 3 [file Table3.DOCX]

**Supplementary Table S3.** Regression analysis summary of diversity (Shannon index) and richness for the whole community and for the major bacterial phyla in the doormat samples from the households having no pets versus the percentage of built area within 200 m radius of the study sites.

| Diversity | R2 | DF | t-value | p-value |
| --- | --- | --- | --- | --- |
| All OTU | 0.28 | 33 | -3.60 | 0.001 |
| Proteobacteria | 0.44 | 31 | -3.87 | 0.0005 |
| Gammaproteobacteria | 0.35 | 33 | -4.18 | 0.0002 |
| Actinobacteria | 0.20 | 32 | -2.28 | 0.0293 |
| Bacteroidetes | 0.12 | 33 | -2.09 | 0.0448 |
| Firmicutes |  |  |  | >0.05 |
| Richness |  |  |  |  |
| All OTU | 0.26 | 33 | -3.26 | 0.00257 |
| Proteobacteria | 0.25 | 31 | -2.80 | 0.00862 |
| Gammaproteobacteria |  |  |  | >0.05 |
| Actinobacteria | 0.29 | 33 | -3.66 | 0.000881 |
| Bacteroidetes | 0.14 | 33 | -2.36 | 0.0244 |
