# Supplementary material for: Urbanization Reduces Transfer of Diverse Environmental Microbiota Indoors
Source: Front Microbiol. 2018 Feb 5;9:84. doi: 10.3389/fmicb.2018.00084 (PMC5808279; doi:10.3389/fmicb.2018.00084)
Supplement: Supplementary file 4 [file Table4.DOCX]

**Supplementary Table S4.** Regression analysis summary of diversity (Shannon index) and richness of the major bacterial taxa in the doormat samples versus the percentage of built area within 200 m radius of the study sites after rarefication to even-sampling depth

| Diversity | Rarefied sequence depth | R^2^ | DF | t-value | p-value |
| --- | --- | --- | --- | --- | --- |
| Proteobacteria | 600 | 0.41 | 48 | -5.81 | <0.001 |
| Alphaproteobacteria | 290 | 0.16 | 48 | -3.03 | 0.003 |
| Betaproteobacteria | 40 | 0.23 | 46 | -2.59 | 0.012 |
| Gammaproteobacteria | 90 | 0.32 | 48 | -4.77 | <0.001 |
| Bacteroidetes | 540 | 0.26 | 43 | -3.901 | <0.001 |
| Firmicutes | 60 | 0.08 | 44 | -1.95 | 0.05 |
| Actinobacteria | 300 | 0.05 | 46 | -1.60 | 0.11 |
|  |  |  |  |  |  |
| Richness |  |  |  |  |  |
| Proteobacteria | 600 | 0.38 | 48 | -5.38 | <0.001 |
| Alphaproteobacteria | 290 | 0.19 | 48 | -3.34 | 0.001 |
| Betaproteobacteria | 40 | 0.25 | 46 | -2.90 | 0.005 |
| Gammaproteobacteria | 90 | 0.28 | 47 | -3.12 | <0.001 |
| Bacteroidetes | 540 | 0.26 | 43 | -3.90 | <0.001 |
| Firmicutes | 60 | 0.08 | 44 | -2.05 | 0.04 |
| Actinobacteria | 300 | 0.04 | 46 | -1.54 | 0.12 |
