# Supplementary material for: Urbanization Reduces Transfer of Diverse Environmental Microbiota Indoors
Source: Front Microbiol. 2018 Feb 5;9:84. doi: 10.3389/fmicb.2018.00084 (PMC5808279; doi:10.3389/fmicb.2018.00084)
Supplement: Supplementary file 5 [file Table5.DOCX]

**Supplementary Table S5.** The stepwise linear regression analysis statistics of the relative abundance of the whole bacterial community and all the major phyla against the land use (explanatory variables) revealing the AIC values for all the tested models. ( perc.built = percentage of built area within 200 m of the study sites, perc.forest = percentage of forest within 200 m of the study sites, perc.trans = percentage of transitional area within 200 m of the study sites).

**Proteobacteria (abundance)**

Call:

Start: AIC=482.63

abundance.Proteobacteria ~

perc.built + perc.forest + perc.trans

Df Sum of Sq RSS AIC

- perc.forest 1 644 663678 480.68

- perc.trans 1 1708 664742 480.76

<none> 663034 482.63

- perc.built 1 67880 730914 485.50

Step: AIC=480.68

abundance.Proteobacteria ~

perc.built + perc.trans

Df Sum of Sq RSS AIC

- perc.trans 1 1709 665387 478.81

<none> 663678 480.68

+ perc.forest 1 644 663034 482.63

- perc.built 1 84909 748586 484.70

Step: AIC=478.81

abundance.Proteobacteria ~

perc.built

Df Sum of Sq RSS AIC

<none> 665387 478.81

+ perc.trans 1 1709 663678 480.68

+ perc.forest 1 645 664742 480.76

- perc.built 1 84524 749911 482.78

> stepP.A$anova

Stepwise Model Path

Analysis of Deviance Table

Initial Model:

abundance.Proteobacteria ~

perc.built + perc.forest + perc.trans

Final Model:

abundance.Proteobacteria ~

perc.built

Step Df Deviance Resid. Df Resid. Dev AIC

1 46 663033.7 482.6279

2 - perc.forest 1 644.0348 47 663677.8 480.6765

3 - perc.trans 1 1709.2148 48 665387.0 478.8051

Residuals:

Min 1Q Median 3Q Max

-283.215 -67.721 -0.656 58.018 305.415

Coefficients:

Estimate Std. Error t value Pr(>|t|)

(Intercept) 397.1371 26.5936 14.934 <2e-16 ***

perc.built 1.4645 0.5931 2.469 0.0171 *

---

Signif. codes: 0 ‘***’ 0.001 ‘**’ 0.01 ‘*’ 0.05 ‘.’ 0.1 ‘ ’ 1

Residual standard error: 117.7 on 48 degrees of freedom

Multiple R-squared: 0.1127, Adjusted R-squared: 0.09423

F-statistic: 6.097 on 1 and 48 DF, p-value: 0.01715

Call:

lm(formula = list.taxa.all.new.names.ALL.final[[3]][[1]]$Proteobacteria ~

perc.built + perc.forest + perc.trans)

Residuals:

Min 1Q Median 3Q Max

-273.650 -65.139 1.636 61.492 295.956

Coefficients:

Estimate Std. Error t value Pr(>|t|)

(Intercept) 414.9452 53.2547 7.792 6.01e-10 ***

perc.built 1.4162 0.6526 2.170 0.0352 *

perc.forest -0.1943 0.9192 -0.211 0.8335

perc.trans -1.1507 3.3429 -0.344 0.7322

---

Signif. codes: 0 ‘***’ 0.001 ‘**’ 0.01 ‘*’ 0.05 ‘.’ 0.1 ‘ ’ 1

Residual standard error: 120.1 on 46 degrees of freedom

Multiple R-squared: 0.1159, Adjusted R-squared: 0.05819

F-statistic: 2.009 on 3 and 46 DF, p-value: 0.1258

**Alphaproteobacteria (abundance)**

Start: AIC=447.54

abundance.alphaproteobacteria ~

perc.built + perc.forest + perc.trans

Df Sum of Sq RSS AIC

- perc.trans 1 18.046 328711 445.55

- perc.forest 1 38.745 328731 445.55

- perc.built 1 139.296 328832 445.56

<none> 328693 447.54

Step: AIC=445.55

abundance.alphaproteobacteria ~

perc.built + perc.forest

Df Sum of Sq RSS AIC

- perc.forest 1 38.714 328749 443.55

- perc.built 1 140.938 328852 443.57

<none> 328711 445.55

+ perc.trans 1 18.046 328693 447.54

Step: AIC=443.55

abundance.alphaproteobacteria ~

perc.built

Df Sum of Sq RSS AIC

- perc.built 1 235.023 328984 441.59

<none> 328749 443.55

+ perc.forest 1 38.714 328711 445.55

+ perc.trans 1 18.014 328731 445.55

Step: AIC=441.59

abundance.alpharoteobacteria ~

perc.built

Df Sum of Sq RSS AIC

<none> 328984 441.59

+ perc.built 1 235.023 328749 443.55

+ perc.forest 1 132.799 328852 443.57

+ perc.trans 1 20.287 328964 443.58

Stepwise Model Path

Analysis of Deviance Table

Initial Model:

abundance.alphaproteobacteria ~

perc.built + perc.forest + perc.trans

Final Model:

abundance.alphaProteobacteria ~

perc.built

Step Df Deviance Resid. Df Resid. Dev AIC

1 46 328692.6 447.5428

2 - perc.trans 1 18.04627 47 328710.7 445.5455

3 - perc.forest 1 38.71353 48 328749.4 443.5514

4 - perc.built 1 235.02299 49 328984.4 441.5871

Residuals:

Min 1Q Median 3Q Max

-172.54 -60.04 -0.04 64.46 180.46

Coefficients:

Estimate Std. Error t value Pr(>|t|)

(Intercept) 239.54 11.59 20.67 <2e-16 ***

---

Signif. codes: 0 ‘***’ 0.001 ‘**’ 0.01 ‘*’ 0.05 ‘.’ 0.1 ‘ ’ 1

Residual standard error: 81.94 on 49 degrees of freedom

Call:

lm(formula = abundance.alpharoteobacteria ~

perc.built + perc.forest + perc.trans)

Residuals:

Min 1Q Median 3Q Max

-168.667 -57.094 -3.483 64.592 180.490

Coefficients:

Estimate Std. Error t value Pr(>|t|)

(Intercept) 241.58387 37.49604 6.443 6.26e-08 ***

perc.built -0.06416 0.45950 -0.140 0.890

perc.forest 0.04766 0.64722 0.074 0.942

perc.trans -0.11829 2.35371 -0.050 0.960

---

Signif. codes: 0 ‘***’ 0.001 ‘**’ 0.01 ‘*’ 0.05 ‘.’ 0.1 ‘ ’ 1

**Betaproteobacteria (abundance)**

Start: AIC=280.33

list.taxa.all.new.names.ALL.final[[3]][[2]]$Betaproteobacteria ~

perc.built + perc.forest + perc.trans

Df Sum of Sq RSS AIC

- perc.built 1 41.43 11640 278.51

- perc.forest 1 188.95 11788 279.14

<none> 11599 280.33

- perc.trans 1 1275.24 12874 283.55

Step: AIC=278.51

list.taxa.all.new.names.ALL.final[[3]][[2]]$Betaproteobacteria ~

perc.forest + perc.trans

Df Sum of Sq RSS AIC

- perc.forest 1 149.41 11790 277.15

<none> 11640 278.51

+ perc.built 1 41.43 11599 280.33

- perc.trans 1 1282.92 12923 281.74

Step: AIC=277.15

list.taxa.all.new.names.ALL.final[[3]][[2]]$Betaproteobacteria ~

perc.trans

Df Sum of Sq RSS AIC

<none> 11790 277.15

+ perc.forest 1 149.41 11640 278.51

+ perc.built 1 1.89 11788 279.14

- perc.trans 1 1277.87 13068 280.29

> stepBeta.A$anova

Stepwise Model Path

Analysis of Deviance Table

Initial Model:

list.taxa.all.new.names.ALL.final[[3]][[2]]$Betaproteobacteria ~

perc.built + perc.forest + perc.trans

Final Model:

list.taxa.all.new.names.ALL.final[[3]][[2]]$Betaproteobacteria ~

perc.trans

Step Df Deviance Resid. Df Resid. Dev AIC

1 46 11598.97 280.3324

2 - perc.built 1 41.4334 47 11640.40 278.5107

3 - perc.forest 1 149.4083 48 11789.81 277.1484

> summary(stepBeta.A)

Call:

lm(formula = list.taxa.all.new.names.ALL.final[[3]][[2]]$Betaproteobacteria ~

perc.trans)

Residuals:

Min 1Q Median 3Q Max

-31.192 -9.329 -1.408 3.961 53.605

Coefficients:

Estimate Std. Error t value Pr(>|t|)

(Intercept) 29.7502 4.6522 6.395 6.24e-08 ***

perc.trans 0.9952 0.4363 2.281 0.027 *

---

Signif. codes: 0 ‘***’ 0.001 ‘**’ 0.01 ‘*’ 0.05 ‘.’ 0.1 ‘ ’ 1

Residual standard error: 15.67 on 48 degrees of freedom

Multiple R-squared: 0.09779, Adjusted R-squared: 0.07899

F-statistic: 5.203 on 1 and 48 DF, p-value: 0.02703

> summary(reg.betaproteo.abu)

Call:

lm(formula = list.taxa.all.new.names.ALL.final[[3]][[2]]$Betaproteobacteria ~

perc.built + perc.forest + perc.trans)

Residuals:

Min 1Q Median 3Q Max

-30.941 -9.740 -0.571 3.773 53.400

Coefficients:

Estimate Std. Error t value Pr(>|t|)

(Intercept) 25.64652 7.04369 3.641 0.000686 ***

perc.built 0.03499 0.08632 0.405 0.687090

perc.forest 0.10525 0.12158 0.866 0.391176

perc.trans 0.99434 0.44215 2.249 0.029352 *

---

Signif. codes: 0 ‘***’ 0.001 ‘**’ 0.01 ‘*’ 0.05 ‘.’ 0.1 ‘ ’ 1

Residual standard error: 15.88 on 46 degrees of freedom

Multiple R-squared: 0.1124, Adjusted R-squared: 0.05451

F-statistic: 1.942 on 3 and 46 DF, p-value: 0.1361

> summary(stepBeta.A)

Call:

lm(formula = list.taxa.all.new.names.ALL.final[[3]][[2]]$Betaproteobacteria ~

perc.trans)

Residuals:

Min 1Q Median 3Q Max

-31.192 -9.329 -1.408 3.961 53.605

Coefficients:

Estimate Std. Error t value Pr(>|t|)

(Intercept) 29.7502 4.6522 6.395 6.24e-08 ***

perc.trans 0.9952 0.4363 2.281 0.027 *

---

Signif. codes: 0 ‘***’ 0.001 ‘**’ 0.01 ‘*’ 0.05 ‘.’ 0.1 ‘ ’ 1

Residual standard error: 15.67 on 48 degrees of freedom

Multiple R-squared: 0.09779, Adjusted R-squared: 0.07899

F-statistic: 5.203 on 1 and 48 DF, p-value: 0.02703

**Gammaproteobacteria (abundance)**

Start: AIC=435.76

abundance.gammaproteobacteria ~

perc.built + perc.forest + perc.trans

Df Sum of Sq RSS AIC

- perc.forest 1 4437 264147 434.61

- perc.trans 1 8572 268282 435.39

<none> 259710 435.76

- perc.built 1 58044 317754 443.85

Step: AIC=434.61

abundance.gammaproteobacteria ~

perc.built + perc.trans

Df Sum of Sq RSS AIC

- perc.trans 1 8579 272726 434.21

<none> 264147 434.61

+ perc.forest 1 4437 259710 435.76

- perc.built 1 82331 346478 446.18

Step: AIC=434.21

abundance.gammaproteobacteria ~

perc.built

Df Sum of Sq RSS AIC

<none> 272726 434.21

+ perc.trans 1 8579 264147 434.61

+ perc.forest 1 4445 268282 435.39

- perc.built 1 81454 354180 445.28

> stepGamma.A$anova

Stepwise Model Path

Analysis of Deviance Table

Initial Model:

abundance.gammaproteobacteria ~

perc.built + perc.forest + perc.trans

Final Model:

abundance.gammaproteobacteria ~

perc.built

Step Df Deviance Resid. Df Resid. Dev AIC

1 46 259709.7 435.7648

2 - perc.forest 1 4437.081 47 264146.8 434.6119

3 - perc.trans 1 8579.423 48 272726.2 434.2100

Residuals:

Min 1Q Median 3Q Max

-119.443 -48.625 -9.269 31.789 184.828

Coefficients:

Estimate Std. Error t value Pr(>|t|)

(Intercept) 110.0978 33.3299 3.303 0.00186 **

perc.built 1.3096 0.4084 3.206 0.00245 **

perc.forest -0.5100 0.5753 -0.887 0.37995

perc.trans -2.5780 2.0922 -1.232 0.22414

---

Signif. codes: 0 ‘***’ 0.001 ‘**’ 0.01 ‘*’ 0.05 ‘.’ 0.1 ‘ ’ 1

Residual standard error: 75.14 on 46 degrees of freedom

Multiple R-squared: 0.2667, Adjusted R-squared: 0.2189

F-statistic: 5.578 on 3 and 46 DF, p-value: 0.002385

**Bacteroidetes (Abundance)**

Start: AIC=495.12

list.taxa.all.new.names.ALL.final[[3]][[1]]$Bacteroidetes ~ perc.built +

perc.forest + perc.trans

Df Sum of Sq RSS AIC

- perc.built 1 150.4 851306 493.13

- perc.trans 1 10878.0 862033 493.75

- perc.forest 1 13954.0 865109 493.93

<none> 851155 495.12

Step: AIC=493.13

list.taxa.all.new.names.ALL.final[[3]][[1]]$Bacteroidetes ~ perc.forest +

perc.trans

Df Sum of Sq RSS AIC

- perc.trans 1 10840.0 862146 491.76

- perc.forest 1 17535.8 868842 492.14

<none> 851306 493.13

+ perc.built 1 150.4 851155 495.12

Step: AIC=491.76

list.taxa.all.new.names.ALL.final[[3]][[1]]$Bacteroidetes ~ perc.forest

Df Sum of Sq RSS AIC

- perc.forest 1 17376.2 879522 490.76

<none> 862146 491.76

+ perc.trans 1 10840.0 851306 493.13

+ perc.built 1 112.4 862033 493.75

Step: AIC=490.76

list.taxa.all.new.names.ALL.final[[3]][[1]]$Bacteroidetes ~ 1

Df Sum of Sq RSS AIC

<none> 879522 490.76

+ perc.forest 1 17376.2 862146 491.76

+ perc.trans 1 10680.4 868842 492.14

+ perc.built 1 3519.7 876002 492.56

> stepBact.A$anova

Stepwise Model Path

Analysis of Deviance Table

Initial Model:

list.taxa.all.new.names.ALL.final[[3]][[1]]$Bacteroidetes ~ perc.built +

perc.forest + perc.trans

Final Model:

list.taxa.all.new.names.ALL.final[[3]][[1]]$Bacteroidetes ~ 1

Step Df Deviance Resid. Df Resid. Dev AIC

1 46 851155.4 495.1164

2 - perc.built 1 150.4039 47 851305.8 493.1252

3 - perc.trans 1 10839.9788 48 862145.8 491.7578

4 - perc.forest 1 17376.2020 49 879522.0 490.7555

> summary(stepBact.A)

Call:

lm(formula = list.taxa.all.new.names.ALL.final[[3]][[1]]$Bacteroidetes ~

1)

Residuals:

Min 1Q Median 3Q Max

-271.00 -71.75 -32.50 70.75 569.00

Coefficients:

Estimate Std. Error t value Pr(>|t|)

(Intercept) 477.00 18.95 25.18 <2e-16 ***

---

Signif. codes: 0 ‘***’ 0.001 ‘**’ 0.01 ‘*’ 0.05 ‘.’ 0.1 ‘ ’ 1

Residual standard error: 134 on 49 degrees of freedom

> summary(reg.bacter.abu)

Call:

lm(formula = list.taxa.all.new.names.ALL.final[[3]][[1]]$Bacteroidetes ~

perc.built + perc.forest + perc.trans)

Residuals:

Min 1Q Median 3Q Max

-273.51 -66.02 -24.65 69.09 547.16

Coefficients:

Estimate Std. Error t value Pr(>|t|)

(Intercept) 526.71790 60.33855 8.729 2.55e-11 ***

perc.built 0.06666 0.73942 0.090 0.929

perc.forest -0.90445 1.04151 -0.868 0.390

perc.trans -2.90411 3.78759 -0.767 0.447

---

Signif. codes: 0 ‘***’ 0.001 ‘**’ 0.01 ‘*’ 0.05 ‘.’ 0.1 ‘ ’ 1

Residual standard error: 136 on 46 degrees of freedom

Multiple R-squared: 0.03225, Adjusted R-squared: -0.03086

F-statistic: 0.511 on 3 and 46 DF, p-value: 0.6767

**Actinobacteria (abundance)**

Start: AIC=454.02

abundance.actinobacteria ~

perc.built + perc.forest + perc.trans

Df Sum of Sq RSS AIC

- perc.trans 1 379 374526 452.07

- perc.forest 1 8598 382745 453.16

<none> 374147 454.02

- perc.built 1 85653 459801 462.33

Step: AIC=452.07

abundance.actinobacteria ~

perc.built + perc.forest

Df Sum of Sq RSS AIC

- perc.forest 1 8600 383126 451.20

<none> 374526 452.07

+ perc.trans 1 379 374147 454.02

- perc.built 1 85493 460019 460.35

Step: AIC=451.2

abundance.actinobacteria ~

perc.built

Df Sum of Sq RSS AIC

<none> 383126 451.20

+ perc.forest 1 8600 374526 452.07

+ perc.trans 1 381 382745 453.16

- perc.built 1 124652 507778 463.29

Stepwise Model Path

Analysis of Deviance Table

Initial Model:

abundance.actinobacteria ~

perc.built + perc.forest + perc.trans

Final Model:

abundance.actinobacteria ~

perc.built

Step Df Deviance Resid. Df Resid. Dev AIC

1 46 374147.5 454.0191

2 - perc.trans 1 378.6745 47 374526.2 452.0697

3 - perc.forest 1 8600.0170 48 383126.2 451.2048

Residuals:

Min 1Q Median 3Q Max

-149.512 -72.263 6.412 68.833 198.512

Coefficients:

Estimate Std. Error t value Pr(>|t|)

(Intercept) 413.580 20.180 20.495 < 2e-16 ***

perc.built -1.778 0.450 -3.952 0.000254 ***

---

Signif. codes: 0 ‘***’ 0.001 ‘**’ 0.01 ‘*’ 0.05 ‘.’ 0.1 ‘ ’ 1

Residual standard error: 89.34 on 48 degrees of freedom

Multiple R-squared: 0.2455, Adjusted R-squared: 0.2298

F-statistic: 15.62 on 1 and 48 DF, p-value: 0.0002538

Call:

lm(formula = abundance.actinobacteria ~

perc.built + perc.forest + perc.trans)

Residuals:

Min 1Q Median 3Q Max

-179.72 -57.85 17.07 59.22 195.86

Coefficients:

Estimate Std. Error t value Pr(>|t|)

(Intercept) 382.4563 40.0048 9.560 1.67e-12 ***

perc.built -1.5909 0.4902 -3.245 0.00219 **

perc.forest 0.7100 0.6905 1.028 0.30926

perc.trans 0.5418 2.5112 0.216 0.83012

---

Signif. codes: 0 ‘***’ 0.001 ‘**’ 0.01 ‘*’ 0.05 ‘.’ 0.1 ‘ ’ 1

Residual standard error: 90.19 on 46 degrees of freedom

Multiple R-squared: 0.2632, Adjusted R-squared: 0.2151

F-statistic: 5.476 on 3 and 46 DF, p-value: 0.002651

**Firmicutes (abundance)**

Start: AIC=532.39

list.taxa.all.new.names.ALL.final[[3]][[1]]$Firmicutes ~ perc.built +

perc.forest + perc.trans

Df Sum of Sq RSS AIC

- perc.forest 1 2591.9 1796224 530.46

- perc.trans 1 5469.2 1799101 530.54

- perc.built 1 30773.1 1824405 531.24

<none> 1793632 532.39

Step: AIC=530.46

list.taxa.all.new.names.ALL.final[[3]][[1]]$Firmicutes ~ perc.built +

perc.trans

Df Sum of Sq RSS AIC

- perc.trans 1 5474 1801698 528.61

- perc.built 1 44058 1840282 529.67

<none> 1796224 530.46

+ perc.forest 1 2592 1793632 532.39

Step: AIC=528.61

list.taxa.all.new.names.ALL.final[[3]][[1]]$Firmicutes ~ perc.built

Df Sum of Sq RSS AIC

- perc.built 1 43545 1845242 527.80

<none> 1801698 528.61

+ perc.trans 1 5474 1796224 530.46

+ perc.forest 1 2596 1799101 530.54

Step: AIC=527.8

list.taxa.all.new.names.ALL.final[[3]][[1]]$Firmicutes ~ 1

Df Sum of Sq RSS AIC

<none> 1845242 527.80

+ perc.built 1 43545 1801698 528.61

+ perc.forest 1 15773 1829469 529.38

+ perc.trans 1 4960 1840282 529.67

> stepFirmi.A$anova

Stepwise Model Path

Analysis of Deviance Table

Initial Model:

list.taxa.all.new.names.ALL.final[[3]][[1]]$Firmicutes ~ perc.built +

perc.forest + perc.trans

Final Model:

list.taxa.all.new.names.ALL.final[[3]][[1]]$Firmicutes ~ 1

Step Df Deviance Resid. Df Resid. Dev AIC

1 46 1793632 532.3865

2 - perc.forest 1 2591.907 47 1796224 530.4587

3 - perc.trans 1 5473.752 48 1801698 528.6108

4 - perc.built 1 43544.678 49 1845242 527.8049

> summary(stepFirmi.A)

Call:

lm(formula = list.taxa.all.new.names.ALL.final[[3]][[1]]$Firmicutes ~

1)

Residuals:

Min 1Q Median 3Q Max

-176.52 -128.02 -81.02 92.48 787.48

Coefficients:

Estimate Std. Error t value Pr(>|t|)

(Intercept) 184.52 27.44 6.724 1.78e-08 ***

---

Signif. codes: 0 ‘***’ 0.001 ‘**’ 0.01 ‘*’ 0.05 ‘.’ 0.1 ‘ ’ 1

Residual standard error: 194.1 on 49 degrees of freedom

> summary(reg.firmi.abu)

Call:

lm(formula = list.taxa.all.new.names.ALL.final[[3]][[1]]$Firmicutes ~

perc.built + perc.forest + perc.trans)

Residuals:

Min 1Q Median 3Q Max

-202.96 -125.54 -76.53 100.85 770.77

Coefficients:

Estimate Std. Error t value Pr(>|t|)

(Intercept) 181.1836 87.5905 2.069 0.0442 *

perc.built 0.9536 1.0734 0.888 0.3790

perc.forest -0.3898 1.5119 -0.258 0.7977

perc.trans -2.0592 5.4983 -0.375 0.7097

---

Signif. codes: 0 ‘***’ 0.001 ‘**’ 0.01 ‘*’ 0.05 ‘.’ 0.1 ‘ ’ 1

Residual standard error: 197.5 on 46 degrees of freedom

Multiple R-squared: 0.02797, Adjusted R-squared: -0.03542

F-statistic: 0.4412 on 3 and 46 DF, p-value: 0.7246
