# Supplementary material for: Urbanization Reduces Transfer of Diverse Environmental Microbiota Indoors
Source: Front Microbiol. 2018 Feb 5;9:84. doi: 10.3389/fmicb.2018.00084 (PMC5808279; doi:10.3389/fmicb.2018.00084)
Supplement: Supplementary file 6 [file Table6.DOCX]

**Supplementary Table S6.** Regression analysis summary of relative abundance of bacterial genus, family, order or class in the doormat samples versus the percentage of built area within 200 m radius of the study sites

| Genus | R^2^ | DF | t-value | p-value |
| --- | --- | --- | --- | --- |
| Streptococcus | 0.12 | 48 | 2.5 | 0.01 |
| Mycobacterium | 0.10 | 47 | 2.01 | 0.049 |
| Solirubrobacter | 0.12 | 48 | -2.06 | 0.01 |
| Arsenicicoccus | 0.16 | 47 | -2.18 | 0.03 |
| Pseudomonas | 0.11 | 48 | -2.46 | 0.01 |
| Family |  |  |  |  |
| Streptococcaceae | 0.10 | 48 | 2.36 | 0.02 |
| Enterobacteriaceae | 0.13 | 48 | 2.63 | 0.01 |
| Mycobacteriaceae | 0.10 | 47 | 2.01 | 0.04 |
| Pseudomonadaceae | 0.22 | 47 | -3.31 | 0.001 |
| Solirubrobacteraceae | 0.12 | 48 | -2.60 | 0.01 |
| Rhodobacteraceae | 0.22 | 47 | -3.61 | <0.001 |
| Planococcaceae | 0.17 | 47 | -2.92 | 0.005 |
| Intrasporangiaceae | 0.31 | 47 | -4.38 | <0.001 |
| Order |  |  |  |  |
| Actinomycetales | 0.21 | 48 | -3.66 | <0.001 |
| Bacillales | 0.22 | 48 | -3.68 | <0.001 |
| Solirubrobacterales | 0.10 | 48 | -2.34 | 0.02 |
| Enterobacteriales | 0.12 | 48 | 2.64 | 0.01 |
| Rhodobacterales | 0.22 | 47 | -3.61 | <0.001 |
| Rubrobacterales | 0.12 | 48 | -2.61 | 0.01 |
| Gp_16 | 0.07 | 48 | -2.03 | 0.04 |
| Class |  |  |  |  |
| Actinobacteria | 0.23 | 48 | -3.86 | <0.001 |
| Acidobacteria_Gp16 | 0.07 | 48 | -2.03 | 0.04 |
