# Supplementary material for: Urbanization Reduces Transfer of Diverse Environmental Microbiota Indoors
Source: Front Microbiol. 2018 Feb 5;9:84. doi: 10.3389/fmicb.2018.00084 (PMC5808279; doi:10.3389/fmicb.2018.00084)
Supplement: Supplementary file 7 [file Table7.DOCX]

**Supplementary Table S7.** Regression analysis summary of the relative abundance of the major bacterial phyla in the doormat samples from the households having no pets versus the percentage of built area within 200 m radius of the study sites.

|  | R2 | DF | t-value | p-value |
| --- | --- | --- | --- | --- |
| Proteobacteria | 0.11 | 31 | 0.85 | 0.402 |
| Gammaproteobacteria | 0.34 | 31 | 3.07 | 0.00442 |
| Actinobacteria | 0.32 | 31 | -3.12 | 0.00392 |
