# Supplementary material for: Urbanization Reduces Transfer of Diverse Environmental Microbiota Indoors
Source: Front Microbiol. 2018 Feb 5;9:84. doi: 10.3389/fmicb.2018.00084 (PMC5808279; doi:10.3389/fmicb.2018.00084)
Supplement: Supplementary file 9 [file Image1.PDF]

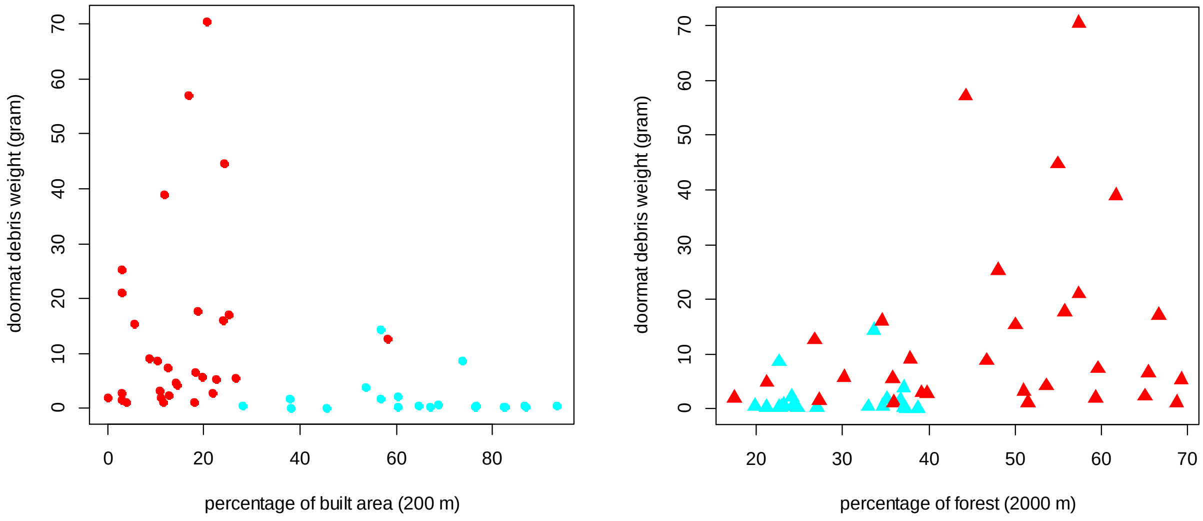

A. **B.**

Supplementary figure S1: Correlation plot of doormat weight (gram) against the land use surrounding the study sites. The litter weight is negatively correlated with the percentage of built area within 200 m (**A**) and positively correlated with the percentage of forest within 2000 m (**B**). The red full circles and triangles represent rural sites and those in sky blue indicate urban sites.
