# Supplementary material for: Urbanization Reduces Transfer of Diverse Environmental Microbiota Indoors
Source: Front Microbiol. 2018 Feb 5;9:84. doi: 10.3389/fmicb.2018.00084 (PMC5808279; doi:10.3389/fmicb.2018.00084)
Supplement: Supplementary file 10 [file Image2.PDF]

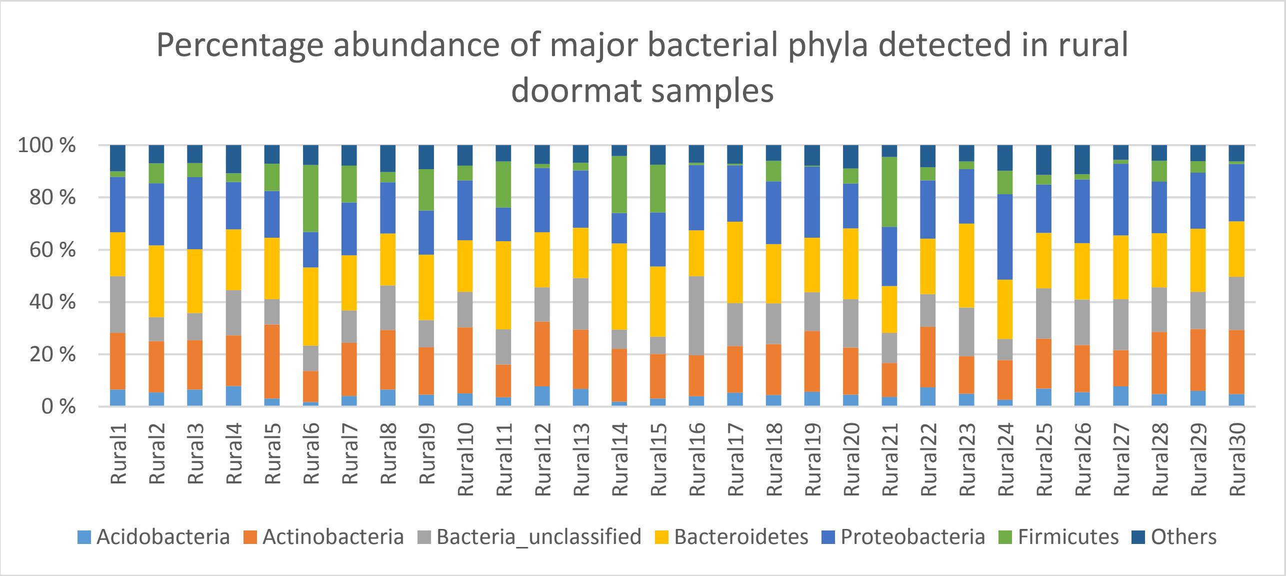

A.

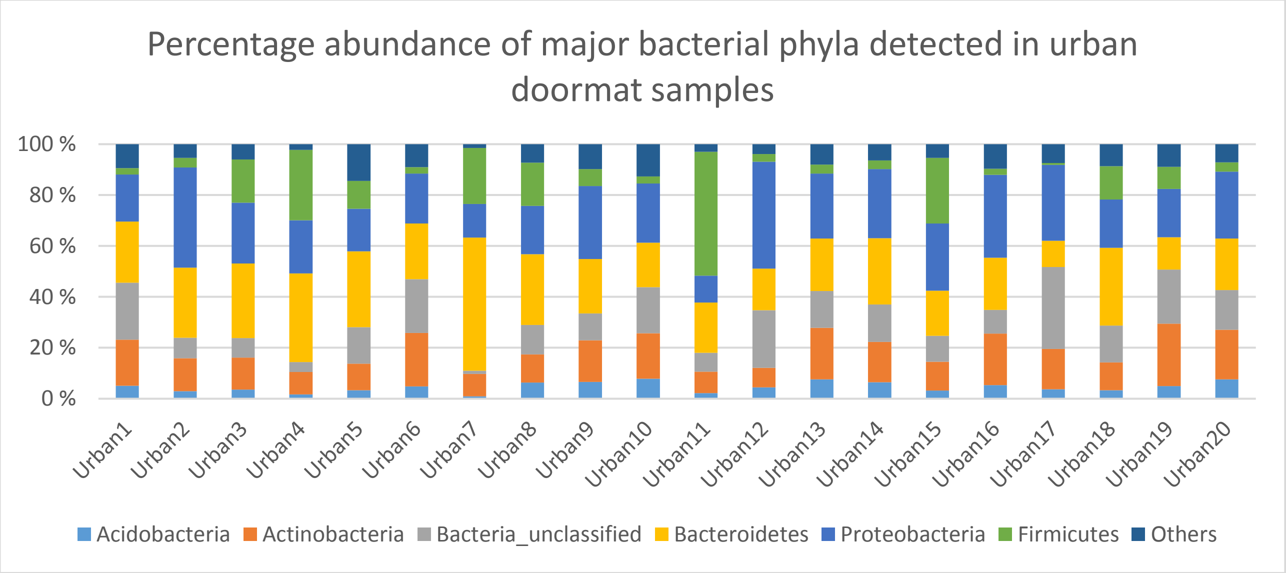

B.

Supplementary figure S2: Distribution of major bacterial phyla in the debris collected from the doormat samples from the rural sites (A) and urban sites (B).
