# Supplementary material for: Urbanization Reduces Transfer of Diverse Environmental Microbiota Indoors
Source: Front Microbiol. 2018 Feb 5;9:84. doi: 10.3389/fmicb.2018.00084 (PMC5808279; doi:10.3389/fmicb.2018.00084)
Supplement: Supplementary file 11 [file Image3.PDF]

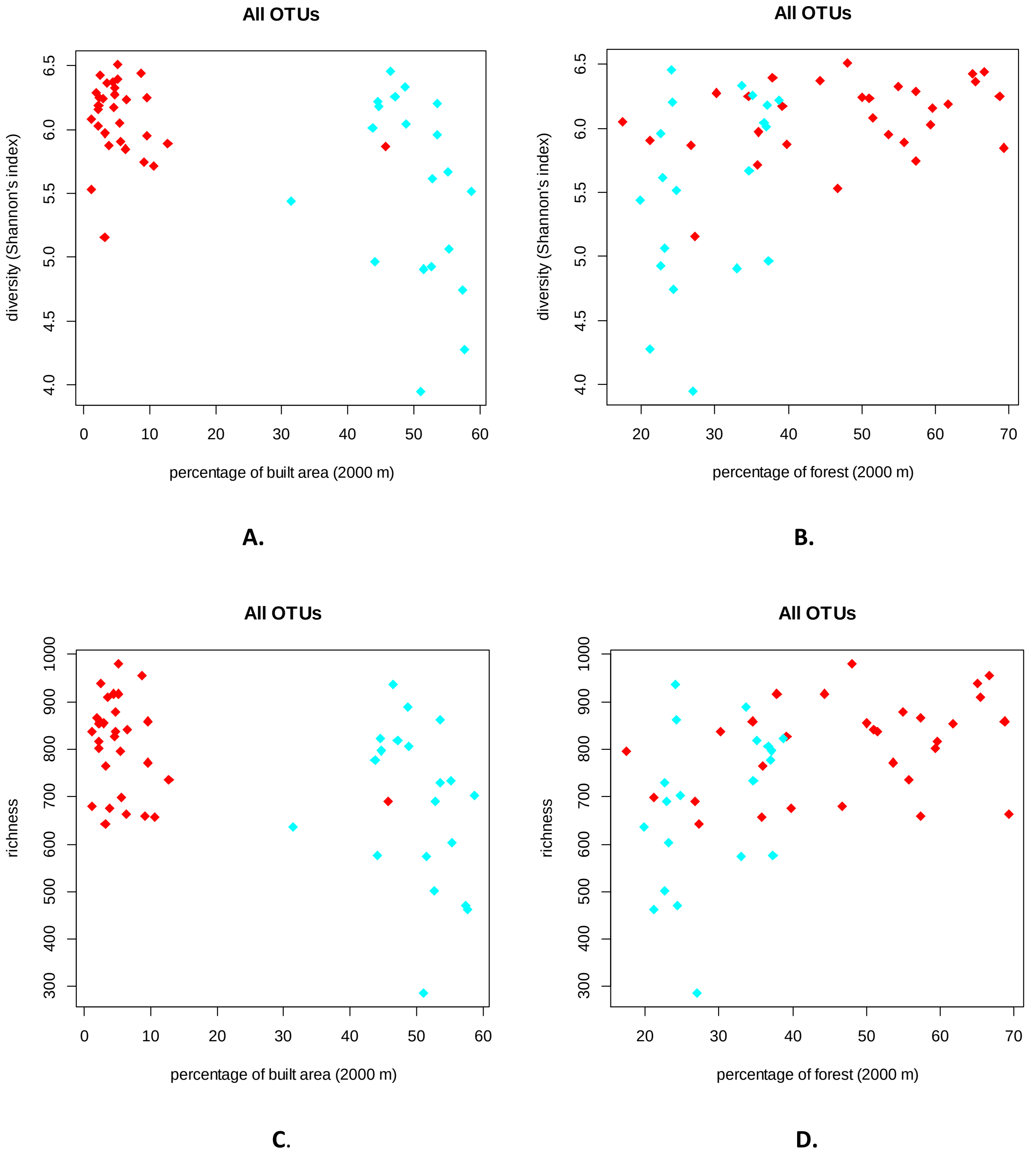

Supplementary figure S3: Correlation plot of Shannon's diversity index of all bacterial community at the OTU level against the percentage of built area (A) and percentage of forest (B) and richness against the percentage of built area (C) and percentage of forest (D) within 2000 m radius of study sites. The red diamonds represent rural sites and the sky blue diamonds represent urban sites.
