# Supplementary material for: Urbanization Reduces Transfer of Diverse Environmental Microbiota Indoors
Source: Front Microbiol. 2018 Feb 5;9:84. doi: 10.3389/fmicb.2018.00084 (PMC5808279; doi:10.3389/fmicb.2018.00084)
Supplement: Supplementary file 12 [file Image4.PDF]

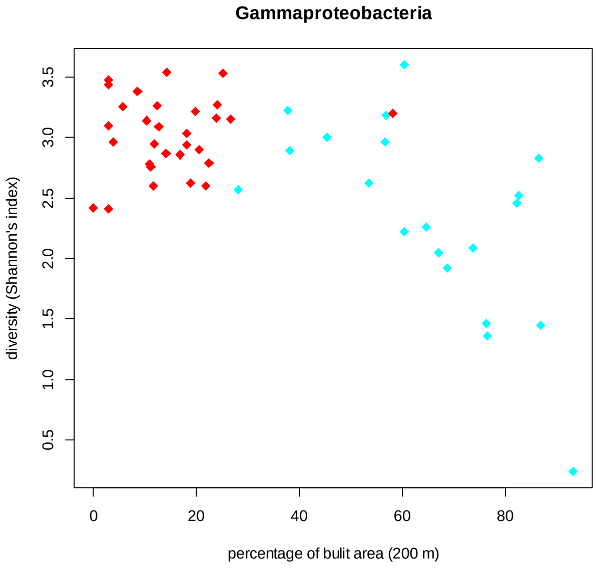
Gammaproteobacteria
diversity (Shannon's index)
0.5
1.0
1.5
2.0
2.5
3.0
3.5
0
20
40
60
80
percentage of bulit area (200 m)

A.

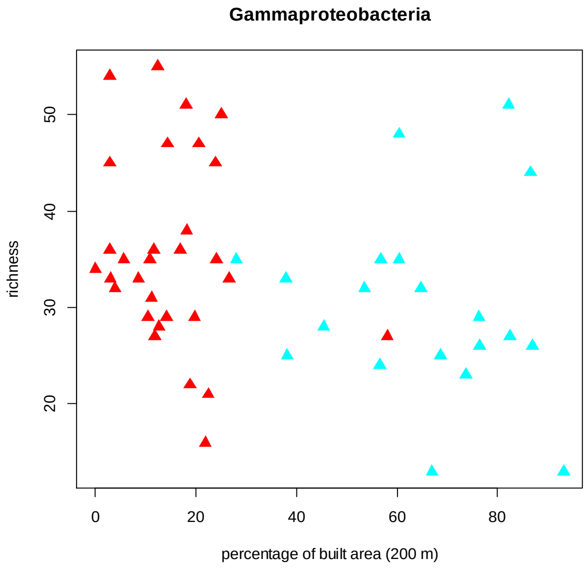
Gammaproteobacteria
richness
20
30
40
50
0
20
40
60
80
percentage of built area (200 m)

B.

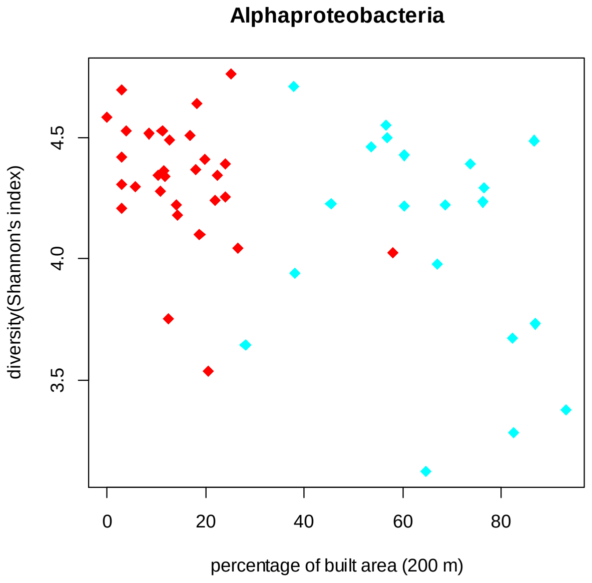
Alphaproteobacteria
diversity(Shannon's index)
3.5
4.0
4.5
0
20
40
60
80
percentage of built area (200 m)

C.

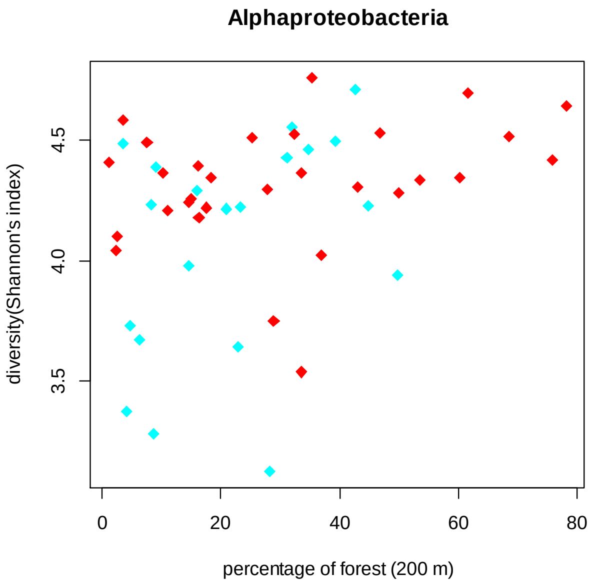
Alphaproteobacteria
diversity(Shannon's index)
3.5
4.0
4.5
0
20
40
60
80
percentage of forest (200 m)

D.

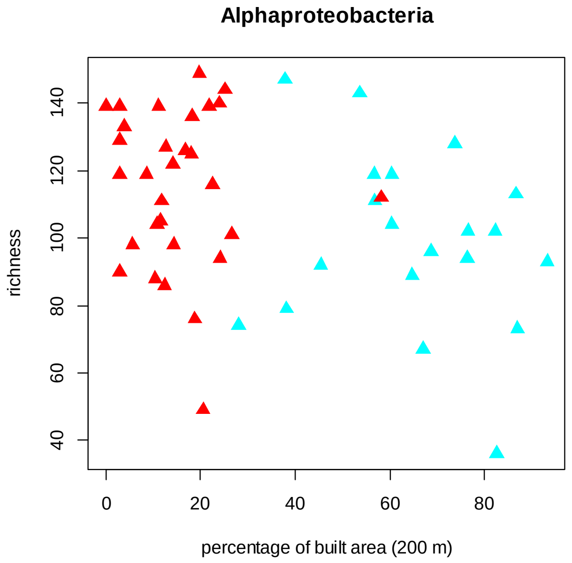

E.

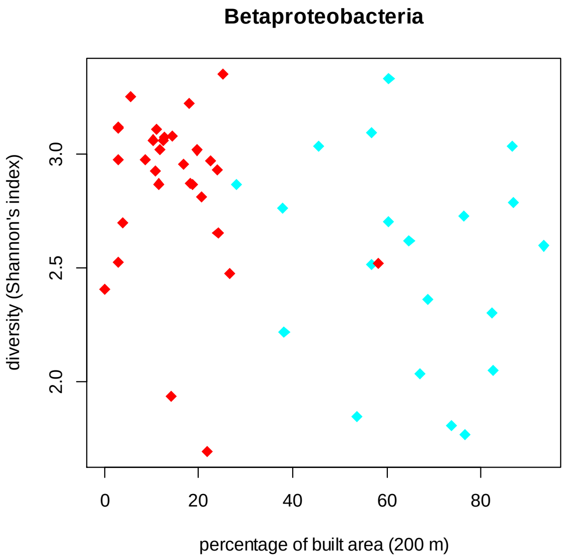

F.

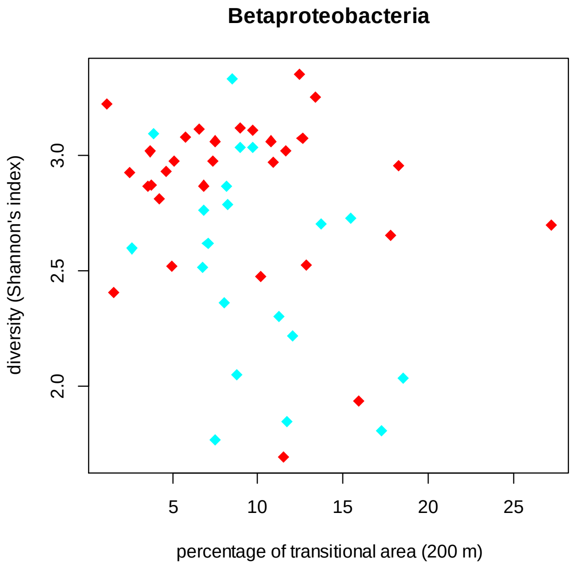

G.

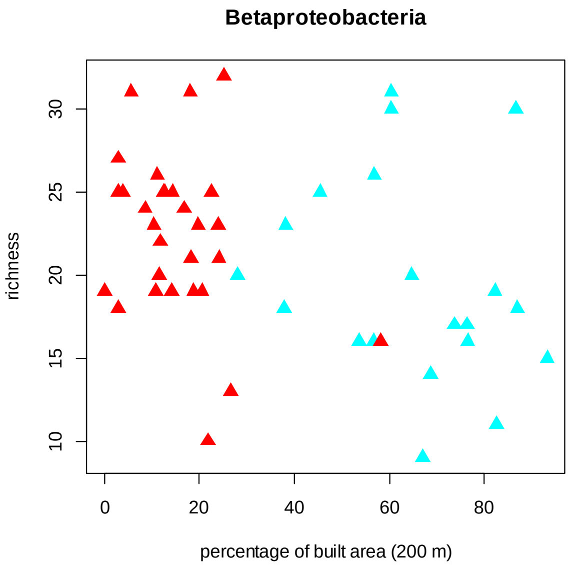

H.

Supplementary figure S4: Correlation plots of diversity index and richness of different classes of Proteobacteria against the land use coverage within 200 m radius of study sites. The Shannon's diversity index (A) and richness (B) of Gammaproteobacteria decrease with the increase in the percentage of built area. The Shannon's diversity index of Alphaproteobacteria decreases with the increase in the percentage of built (C) but increases with the increase in the percentage of forest (D) while its richness decreases against the percentage of built area (E).

The Shannon's diversity index of Betaproteobacteria decreases with the increase in the percentage of built area (F) and percentage of transitional area (G) and its richness decreases with an increase in the percentage of built area (H). The red diamonds and triangles represent rural sites and the square blue ones represent urban sites.
