# Supplementary material for: Urbanization Reduces Transfer of Diverse Environmental Microbiota Indoors
Source: Front Microbiol. 2018 Feb 5;9:84. doi: 10.3389/fmicb.2018.00084 (PMC5808279; doi:10.3389/fmicb.2018.00084)
Supplement: Supplementary file 13 [file Image5.PDF]

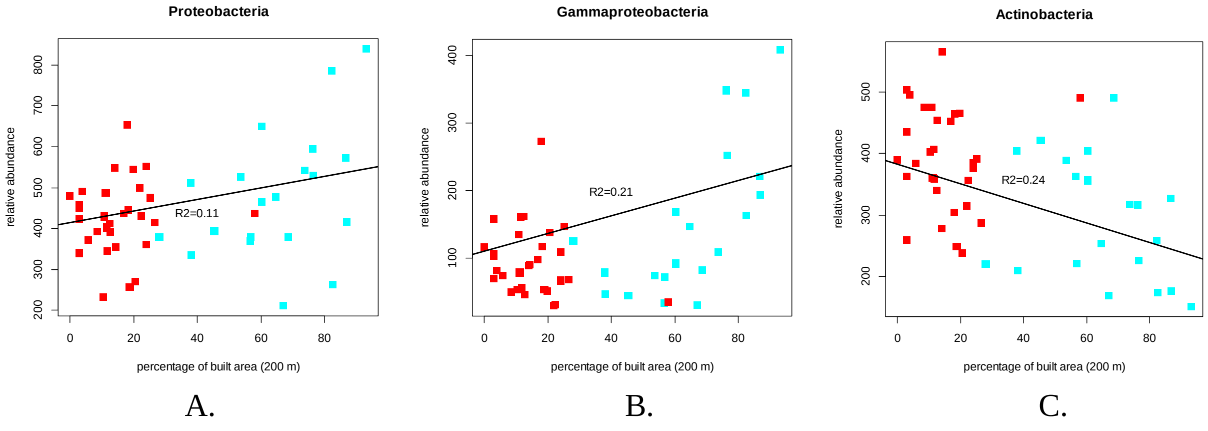

Supplementary Figure S5. **The relative abundance of major bacterial phyla in doormat debris in relation to the percentage of built area within 200 m radius surrounding person's home**. The relative abundance of Proteobacteria (A) and its class Gammaproteobacteria (B) increased while the relative abundance of Actinobacteria (C) decreased with the increase in the percentage of built area. The red squares represent the rural sites and the blue squares denote urban sites.
