# Supplementary figures and images for: Urbanization Reduces Transfer of Diverse Environmental Microbiota Indoors
Source: Front Microbiol. 2018 Feb 5;9:84. doi: 10.3389/fmicb.2018.00084 (PMC5808279; doi:10.3389/fmicb.2018.00084)

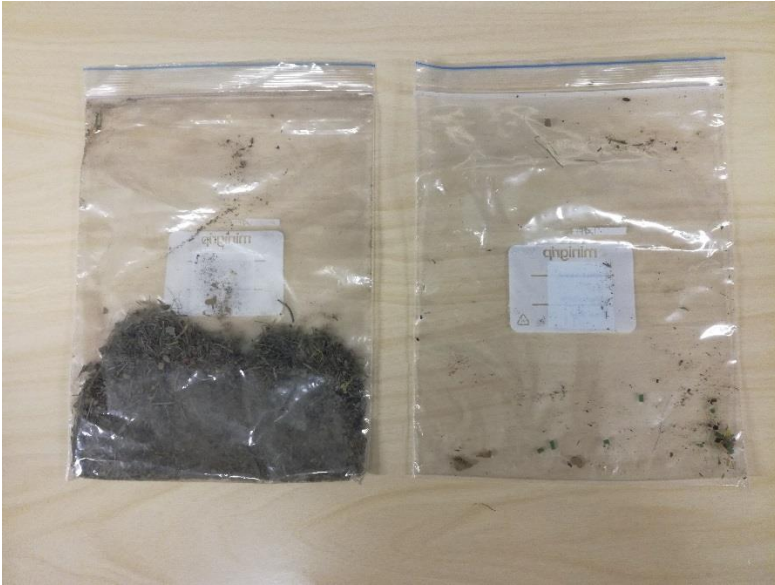

**Supplementary figure S6.** Examples of rural (on the left) and urban (on the right) doormat samples.

Supplement: Supplementary file 14 [file Image6.PDF]
